# Delivery of 3-O-Ethyl-L-Ascorbic Acid from Radiation-Crosslinked Hydrogels to Promote Extracellular Matrix Production in Fibroblasts

**DOI:** 10.3390/ijms27209028

**Published:** 2026-10-12

**Authors:** Bozena Rokita, Agnieszka Adamus-Wlodarczyk, Alicja K. Olejnik, Renata Czechowska-Biskup, Beata Skibska, Slawomir Kadlubowski, Piotr Ulanski, Radoslaw A. Wach

**Affiliations:** 1 BioMatGel Sp. z o.o., Pienista 41F/11, 94-109 Lodz, Polandslawomir.kadlubowski@p.lodz.pl (S.K.); piotr.ulanski@p.lodz.pl (P.U.); 2 Institute of Applied Radiation Chemistry, Faculty of Chemistry, Lodz University of Technology, Wroblewskiego 15, 93-590 Lodz, Poland; 3 Department of Applied Pharmacy, Faculty of Pharmacy, Medical University of Lodz, Muszynskiego 1, 90-151 Lodz, Poland

**Keywords:** hydrogels, radiation crosslinking, ethyl ascorbic acid, vitamin C, active substance release, cytocompatibility

## Abstract

L-ascorbic acid is a bioactive compound widely used in dermatology and cosmetology, owing to its antioxidant properties and essential role in collagen biosynthesis. However, its poor chemical stability in aqueous formulations considerably limits its incorporation into hydrogel-based delivery systems. In the present study, a radiation-crosslinked hydrogel based on poly(N-vinylpyrrolidone), poly(ethylene glycol) and agar was developed as a carrier for 3-O-ethyl-L-ascorbic acid (EvitC), a stabilized vitamin C derivative, for topical application. The influence of EvitC on hydrogel formation, physicochemical properties, release characteristics and long-term stability was investigated. In parallel, the biological safety and activity of EvitC and EvitC-containing hydrogel were evaluated using human dermal fibroblasts. Cell metabolic activity was assessed using the XTT assay and further supported by the qualitative evaluation of cell viability using Live/Dead staining, while extracellular matrix production was assessed using the Col-F assay. Hydrogels containing 0.1% EvitC exhibited advantageous essential properties, such as gel fraction, swelling capacity and mechanical properties while maintaining the sustained release of the biologically active compound. The components of the hydrogel matrix effectively protected EvitC during radiation processing and storage, thereby confirming the overall stability of the formulation. Neither EvitC nor hydrogel extracts induced cytotoxic effects under all tested conditions. Furthermore, the prolonged exposure of fibroblasts to hydrogel extracts resulted in a moderate but statistically significant increase in ECM-associated Col-F, i.e., collagen- and elastin-associated fluorescence. These findings support the potential of the developed radiation-crosslinked hydrogel as a stable and cytocompatible platform for the topical delivery of EvitC. The combination of favorable physicochemical properties, formulation stability and excellent biological performance highlights its potential for the development of advanced hydrogel-based skin care formulations.

## 1. Introduction

L-ascorbic acid or vitamin C (vitC), a water-soluble vitamin essential for human health, is a critical component in maintaining the physiological function and structural integrity of the skin [1,2,3,4]. As the body’s largest organ, the skin provides the primary protective barrier against harmful environmental factors, and its condition is closely linked to nutritional status. Humans are incapable of synthesizing vitC endogenously, making dietary intake the sole source for maintaining adequate tissue levels [5]. Normal skin naturally contains high concentrations of vitC, with the epidermis holding more than the dermis, which is acquired from the bloodstream. This reservoir supports a multitude of vital functions, but it is susceptible to reduction resulting from various stressors, predominantly exposure to ultraviolet (UV) radiation and the natural aging process [2,6].

The dermatological significance of vitamin C stems from its versatile biological functions. It is an efficient antioxidant, an essential cofactor in collagen synthesis, a modulator of inflammatory pathways, and an inhibitor of melanogenesis [2,5,7,8]. These mechanisms emphasize both its medical and cosmetic value. In modern cosmetic science, vitC has become one of the most widely exploited ingredients, featured prominently in serums, creams, and other skin care formulations aimed at improving skin quality, brightness, and firmness [7,9,10]. Its significance is supported by extensive scientific evidence demonstrating its broad benefits for skin appearance and health [4,7,11].

The most important function of vitamin C in the skin is its supporting but indispensable role in collagen synthesis, the primary structural protein in the dermis that provides the skin with its strength and resilience [2]. VitC acts as one of the factors for the enzymes prolyl and lysyl hydroxylase, which are responsible for hydroxylating proline and lysine residues during procollagen synthesis [6,8]. This hydroxylation step is important for further stabilization of the collagen triple helix structure. Consequently, a deficiency in vitC leads to reduced collagen production, manifesting clinically as compromised wound healing, increased capillary fragility, and the classic cutaneous signs of scurvy [8]. Studies have shown that vitC preferentially enhances the gene transcription of type I and III collagen [12]. Clinical trials on the topical application of vitC confirmed this biochemical function, demonstrating new collagen formation in biopsied skin, which in turn correlates also with improvements in skin wrinkling and overall appearance [13].

Beyond its structural role, vitamin C is arguably the most important non-enzymatic, water-soluble antioxidant in the skin [4,14]. The skin is constantly exposed to reactive oxygen species (ROS), or free radicals, generated from both endogenous metabolic processes and external sources like UV radiation and environmental pollution [15,16]. These detrimental factors inflict oxidative stress, causing widespread damage to cellular components, including DNA, lipids, and proteins [17,18]. This cumulative damage is a primary driver of both intrinsic skin aging and extrinsic photoaging, and it contributes to the pathogenesis of inflammatory skin diseases and skin cancer [2,8,19]. VitC effectively neutralizes free radicals, thereby protecting the skin from oxidative damage. This photoprotective effect is one of a key aspects of its applicability in dermatology [20,21]. Although vitamin C does not absorb UV light, its topical application has been shown to limit acute UV-induced damage, such as the formation of sunburn cells, and to reduce DNA mutations like thymine dimers [2,11,19,22]. VitC also plays a significant role in regulating skin pigmentation. It functions as a depigmenting agent by interacting with the enzyme tyrosinase, the rate-limiting step in melanin production [5,23]. By inhibiting tyrosinase activity, vitC effectively reduces the synthesis of melanin, making it a valuable therapeutic agent for treating disorders of hyperpigmentation, such as melasma and post-inflammatory hyperpigmentation [5,24,25].

Despite its well-documented benefits, the effective delivery of L-ascorbic acid into the skin presents significant challenges for formulation technologists [14,23,26]. The primary obstacle is its inherent instability; this water-soluble vitamin degrades rapidly when exposed to air, light, and heat [26,27,28]. Furthermore, its hydrophilic and charged nature hinders its ability to penetrate the lipophilic stratum corneum, the outermost layer of the skin that acts as an effective barrier [29,30,31,32]. Even under optimal conditions, achieving a therapeutic concentration in the dermis remains a challenge. Consequently, much research has focused on developing advanced delivery systems that can enhance the stability and bioavailability of vitC and its derivatives [27,29,31].

Examples of physical methods aiming to temporarily disrupt the stratum corneum, creating pathways for molecules to pass through, includes microdermabrasion [33,34], microneedles [29,34], laser assistance [35,36] or non-invasive iontophoresis [37,38]. On the other hand, formulation-based approaches focus on the incorporation of vitamin C in carriers to protect it from degradation and facilitate its passage into the skin. For instance, the encapsulation of vitC in nano-sized vesicles, such as spanlastics, which are deformable nanovesicles able to immobilize vitC and provide both physical and chemical stability for extended periods; they also improve drug penetration into the stratum corneum [27,29,39]. Another approach is the use of solid-in-oil nanodispersions, where nano-sized particles of hydrophilic vitC are dispersed in an oil vehicle with the help of surfactants [29]. This system enhanced stability and has been shown to improve skin permeation, applied for protection against photoaging. Topical carriers may also be based on fibrous materials, such as electrospun fibers. Dissolvable topical formulation of poly(vinyl alcohol) (PVA) nanofibers loaded with vitC provides a burst release profile [40,41]. A composite system combining PVA nanofibers with β-cyclodextrin molecular capsules can deliver vitC at a constant, zero-order rate, which may be preferable for therapeutic management. A more sophisticated method involves physical-supramolecular hydrogels that can significantly enhance the permeation of both ascorbic acid and its precursor, ascorbyl glucoside [39]. These hydrogels can also preserve the precursor compound until it reaches the target epidermal layer, where it is then converted into active ascorbic acid. More generally, hydrogels are good carriers for bioactive agents, particularly water-soluble molecules, as the nature of hydrogels is hydrophilic [42,43].

The hydrogel comprises a polymer network filled with water. Typically, synthetic hydrophilic polymers are used, and the water content in a hydrogel may reach over 95% and may serve as a matrix or carrier for bioactive compounds [44]. The network is formed through crosslinking, which may be achieved by several chemical methods. Among those, radiation stands as a clean and economical method, as it does not require additional crosslinking agents, initiators, or heating of the reaction mixture to initiate the reaction [45]. Here, the ionizing radiation provides the energy necessary to initiate chemical reactions, specifically radical reactions. Radicals formed along the polymer chains recombine to create covalent bonds linking the chains and resulting in the formation of a chemical gel. The efficiency of crosslink formation depends on numerous factors, including composition, concentrations, additives and irradiation conditions.

The aim of this study was to develop a hydrogel-based delivery system for ascorbic acid by incorporating a stabilized vitamin C derivative into the hydrophilic polymer matrix. As the native L-ascorbic acid is inherently unstable, the work focused also on establishing optimal procedures to ensure its chemical stability throughout the radiation process. The efforts sought to enable the effective release of vitC using a radiation-crosslinked hydrogel carrier and to evaluate the in vitro biological response to the released compound in human dermal fibroblasts, including cytocompatibility and ECM-associated collagen and elastin deposition. The proposed approach is advantageous through its simple one-pot fabrication process, with the incorporated drug providing simultaneous product sterilization.

## 2. Results and Discussion

### 2.1. Impact of Ethyl Ascorbic Acid on Hydrogels Formation

**Figure 1:**
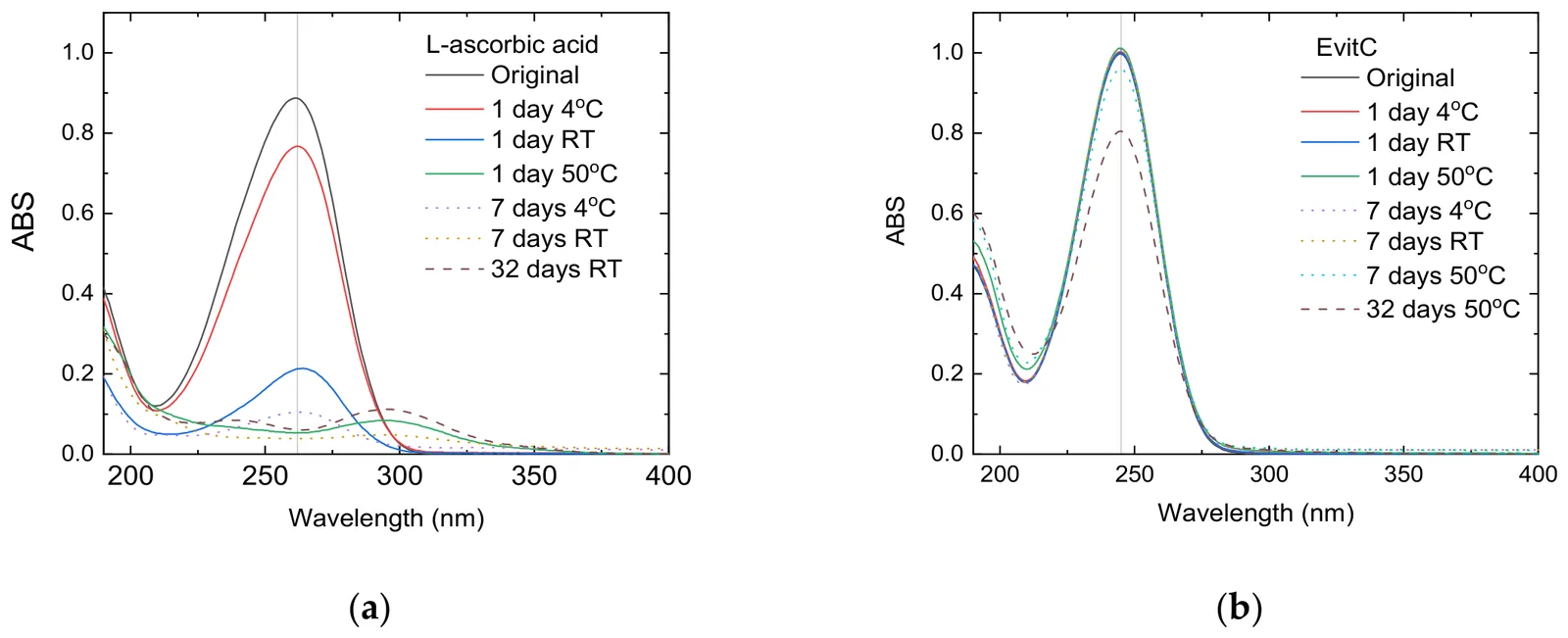
Stability comparison of (**a**) native vitC (L-ascorbic acid) and (**b**) EvitC (3-O-ethyl-L-ascorbic acid) in aqueous solution stored at various temperatures—UV-Vis spectra. Vertical lines denotes wavelengths of maximum absorption of respective compounds.

The native vitamin C is essentially unstable in aqueous environments—such as those characteristic for hydrogels—and undergoes degradation during storage through hydrolysis and upon exposure to light [26], as well as decomposition initiated by ionizing radiation. Besides identifying ascorbic acid derivatives (among native vitamin C, i.e., L-ascorbic acid, ascorbyl glucoside, Na-ascorbyl phosphate, ethyl ascorbic acid) with improved resistance to these destabilizing factors, the initial work focused also on establishing the conditions ensuring the effective formation of hydrogels in the presence of ascorbic acid, which is also a critical issue. Figure 1 presents the exemplary results of the stability screening of native vitamin C and ethyl vitamin C (3-O-ethyl-L-ascorbic acid) in aqueous solution stored at various temperatures. Apparently, the native form decomposes instantly, even if stored refrigerated, whereas ethyl-stabilized ascorbic acid maintains its structure even at elevated temperatures. Therefore, 3-O-ethyl-L-ascorbic acid was employed in all experiments, and termed EvitC hereinafter.

An elemental prerequisite for the safe carrier of bioactive substances is that the material applied to the injury site, compromised skin or intact skin—depending on the intended application, respectively for the wound, abrasion or aged, dry skin (in cosmetology)—should be characterized by a simple composition, free of potentially risky additives. Well-established medical materials are hydrogels, intended for wound healing. Particularly important in wound-healing strategies are hydrogels manufactured with radiation technology, where the recombination of macroradicals, that is the crosslinking of hydrophilic polymers, results in a three-dimensional network—the product is sterilized simultaneously. This technology was commercialized more than three decades ago and is still in use, mainly due to clinical effectiveness of the product and the economic competitiveness [45]. The matrix is biocompatible; therefore, when used for cosmetics, it may be primarily intended for people with dermal problems, sensitive skin and a tendency to allergies. Indeed, the hydrogels formulated in this work contain no auxiliary additives, which is common for cosmetic formulations, but only the active substance of EvitC. This simplified composition was intentionally selected to minimize the number of formulation components while taking advantage of the improved chemical stability of ethyl ascorbic acid [46,47]. Therefore, the hydrogel is anticipated to act in the intended direction, for instance skin regeneration when used as cosmetics, presumably without adverse side effects even for sensitive skin.

**Figure 2:**
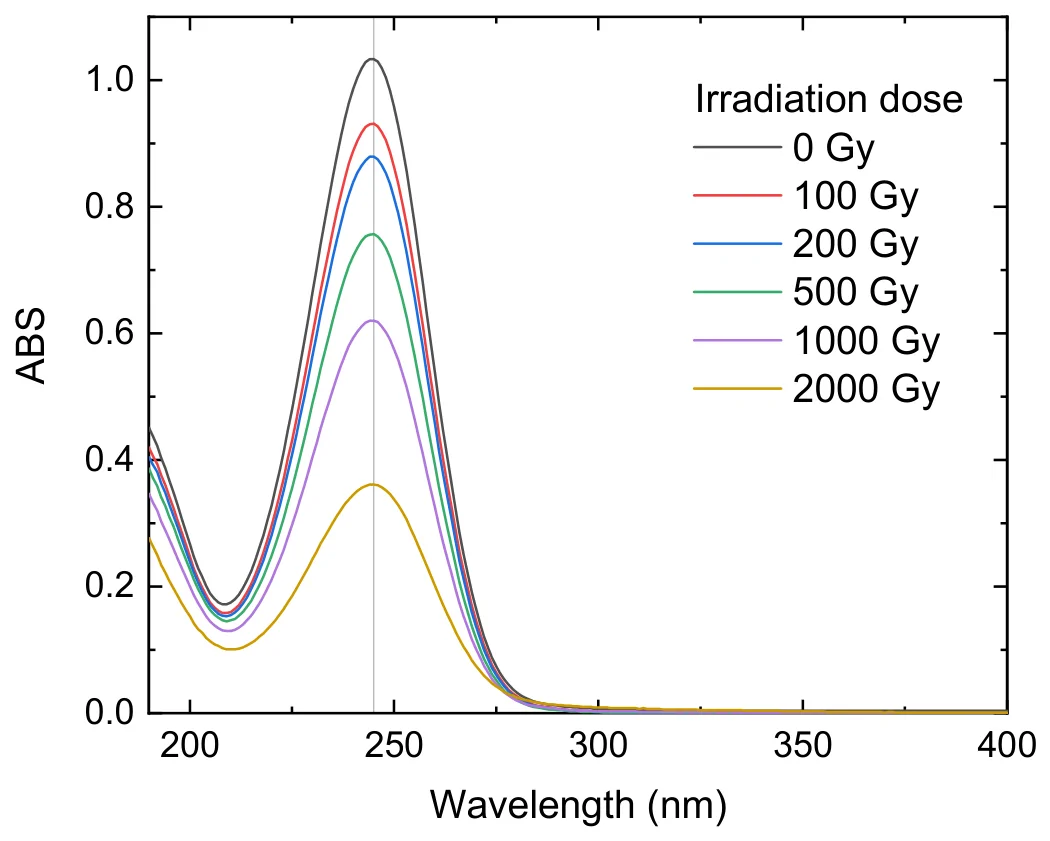
Stability of EvitC (3-O-ethyl-L-ascorbic acid) irradiated by electron beam (EB) up to 2 kGy in 0.002% aqueous solution—UV-Vis spectra, maximum absorption at 245 nm indicated with a vertical line.

The two research issues related to the use of ionizing radiation were to identify the stability of EvitC exposed to ionizing radiation, and to evaluate the influence of EvitC on hydrogel formation. EvitC is an effective radical scavenger; thus, it is unstable when irradiated in solution. This was observed in the present study—irradiation dose of as low as 2 kGy decomposed ca. 70% of EvitC irradiated in 0.002% aqueous solution (Figure 2). The second-order rate constant of the reaction between the hydroxyl radical (^•^OH), the main reactive water radiolysis product, and EvitC is k_(OH + EvitC)_ = 1.1 × 10^10^ M^−1^ s^−1^, which is at a diffusion-controlled level [48,49]. Rate constants of EvitC reactions with hydrogen atom and hydrated electron are two and three orders of magnitude lower, respectively, due to the much less electrophilic (former) and nucleophilic (latter) natures of these species [50]. Due to the fast addition reactions of ^•^OH, several kinds of radicals may be formed on the L-ascorbic acid molecule. Formation of a radical at the ethyl side group via hydrogen abstraction is somewhat less probable [51,52]. The ethyl substitution may lower the rate relative to L-ascorbic acid by removing one reactive hydroxyl site and altering electron density or simply due to steric hindrance, but it is unlikely to move the reaction out of the diffusion-controlled regime. Further, decomposition of L-ascorbic acid has not been elucidated, but it is known that the primary degradation pathway for vitC is oxidation. The ethyl group at the C-3 position slows this process but does not prevent it. Apparently, dehydro ethyl ascorbic acid is formed, which may further hydrolyze, similarly to the native molecule, to form an ethyl derivative of diketogulonic acid. Since 2,3-diketogulonic acid derived from native vitC is biologically inactive [53], it may be hypothesized that the EvitC decomposition product is likewise biologically inactive. This should be treated as a structure-based estimate, not a measured value, which has not yet been determined. Nevertheless, we have demonstrated that EvitC irradiated in aqueous solution at low concentrations undergoes instant and practically complete decomposition, even at relatively low doses.

Data presented in Figure 2 allow for a rough estimation of the radiation-chemical yield of decay of EvitC. This yield is defined as the number of moles of the substance destroyed by one joule of ionizing radiation energy absorbed by the irradiated system. Extrapolation of this yield to the zero dose results in the value ca. 1–2 × 10^−7^ mol J^−1^, i.e., close to the expected radiation-chemical yield of generation of ^•^OH radicals upon the irradiation of water (2.8 × 10^−7^ mol J^−1^). This indicates that, when EvitC is irradiated in water with no other compounds, most of the ^•^OH radicals generated in the solution attack the EvitC molecules, leading to its efficient destruction. Apparently, EvitC will lose its biological activity after its irradiation without other components present in the system.

To form a polymeric network from synthetic hydrophilic polymers having no unsaturated bonds, a relatively high instant concentration of macroradicals is required. This is because biradical reaction, i.e., recombination of macroradicals, is the mechanism of PVP crosslinking. Macroradicals are formed through hydrogen abstraction from polymer chains, principally carbon-bound H, by hydroxyl radicals (k_(OH + PVP)_ = (1.5–2.3) × 10^8^ M^−1^ s^−1^; k_(OH + PEG)_ = 10^8^–10^9^ M^−1^ s^−1^ depending on the molecular weight) [54] and hydrogen atoms produced, next to the hydrated electron, in water radiolysis. The yields of all transient and molecular products of water radiolysis are well known [55]. H^•^ is produced in five-fold-less numbers than hydroxyl radical, and, considering the two-orders-of-magnitude-lower reaction constant indicated above, hydrogen atom’s role in macroradical creation is apparently low. Hydrogels based on PVP, agar and PEG were investigated earlier [56]; therefore, the methodology was reproduced accordingly, yet the composition in the current study is original. Hydrogels were manufactured by EB irradiation of the reaction mixture—PVP 6%, PEG 2%, agar 1% and EvitC in aqueous solution—with a dose of 25 kGy. The main two factors characterizing hydrogel, the gel fraction (GF) and the degree of swelling at equilibrium (EDS), are presented in Table 1. The results for samples with no EvitC—relatively high GF and moderate swelling—are comparable with the literature values for hydrogels of similar compositions [56]. These indicate the high efficiency of crosslinking, which prevails over the degradation that occurs to a limited extent.

**Figure 3:**
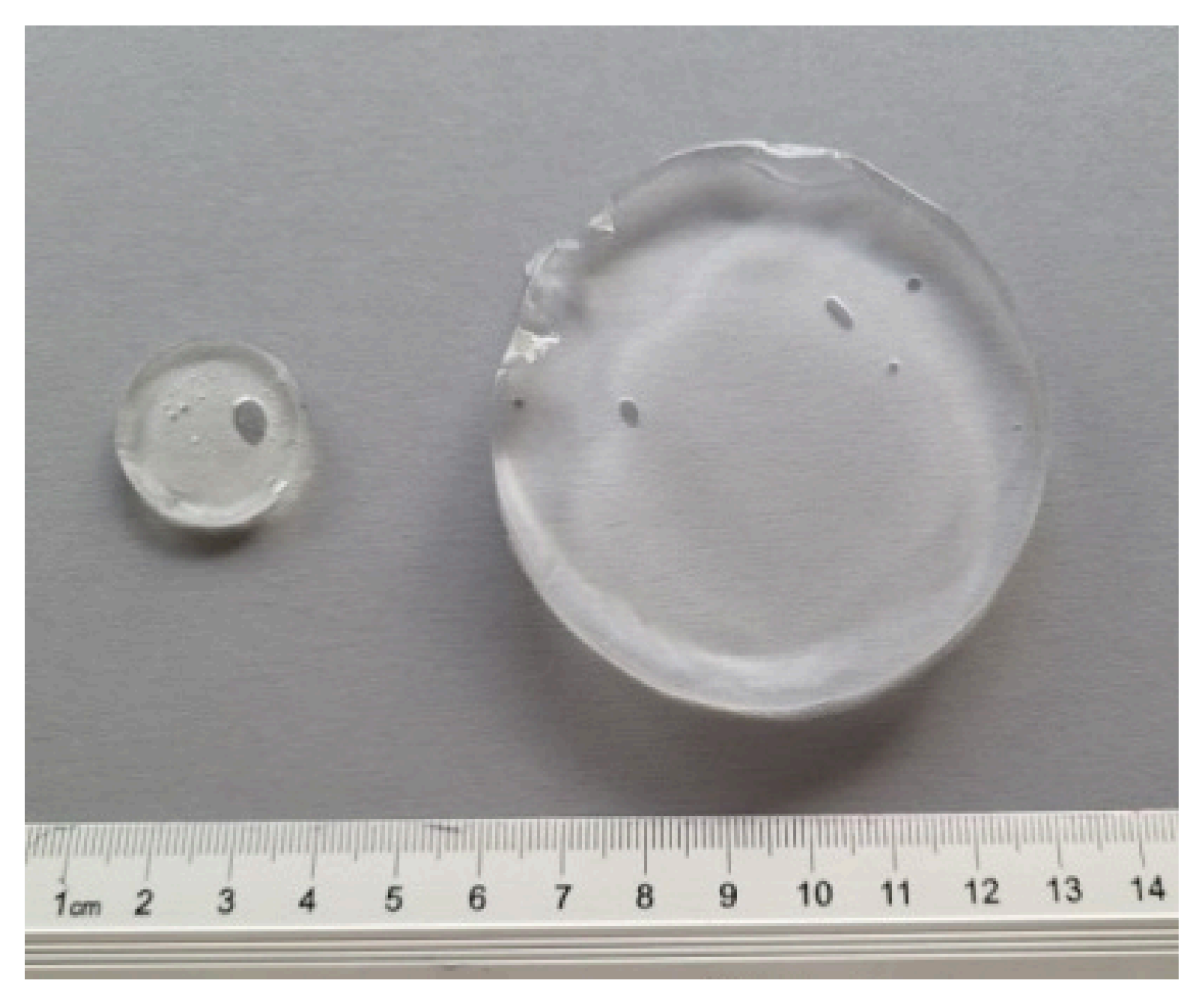
Hydrogel swelling—example hydrogel with 0.05% EvitC after synthesis (**left**) and swollen after 24 h in water (**right**).

The effect of EvitC on gel formation is presented in Table 1. The EvitC scavenger reduces the number of water radiolysis transient products—chiefly only ^•^OH to count—available for reaction with other solutes, and consequently diminishes the number of radicals on macromolecules. This in turn results in a lower yield of PVP crosslinking and lesser gel fraction in the hydrogel. The hydrogel parameters are controlled also by the second factor. The effect depends on the concentration of the scavenger with respect to other components, and to the PVP in particular, i.e., on the EvitC/PVP ratio. As the concentration of PVP, and two other macromolecular components, is constant, the increase in EvitC concentration indirectly but effectively reduces the probability of PVP macroradical recombination. A decrease in GF demonstrates reduction in the radiation yield of crosslinking. Simultaneously, an increase in EDS proves the lower crosslinking density, as the network is looser, capable of absorption of a larger amount of water—an example of swelling of the hydrogel with 0.05% EvitC is presented in Figure 3. A total of 0.5% of EvitC obstructs hydrogel formation, even when 10% of PVP was used—more macroradicals are formed due to reduced ratio of EvitC/PVP, than in the case of 6% PVP composition. Therefore, the upper concentration of the active compound is limited, as it influences the properties of hydrogels.

### 2.2. Mechanical Properties of Hydrogels Containing EvitC

**Figure 4:**
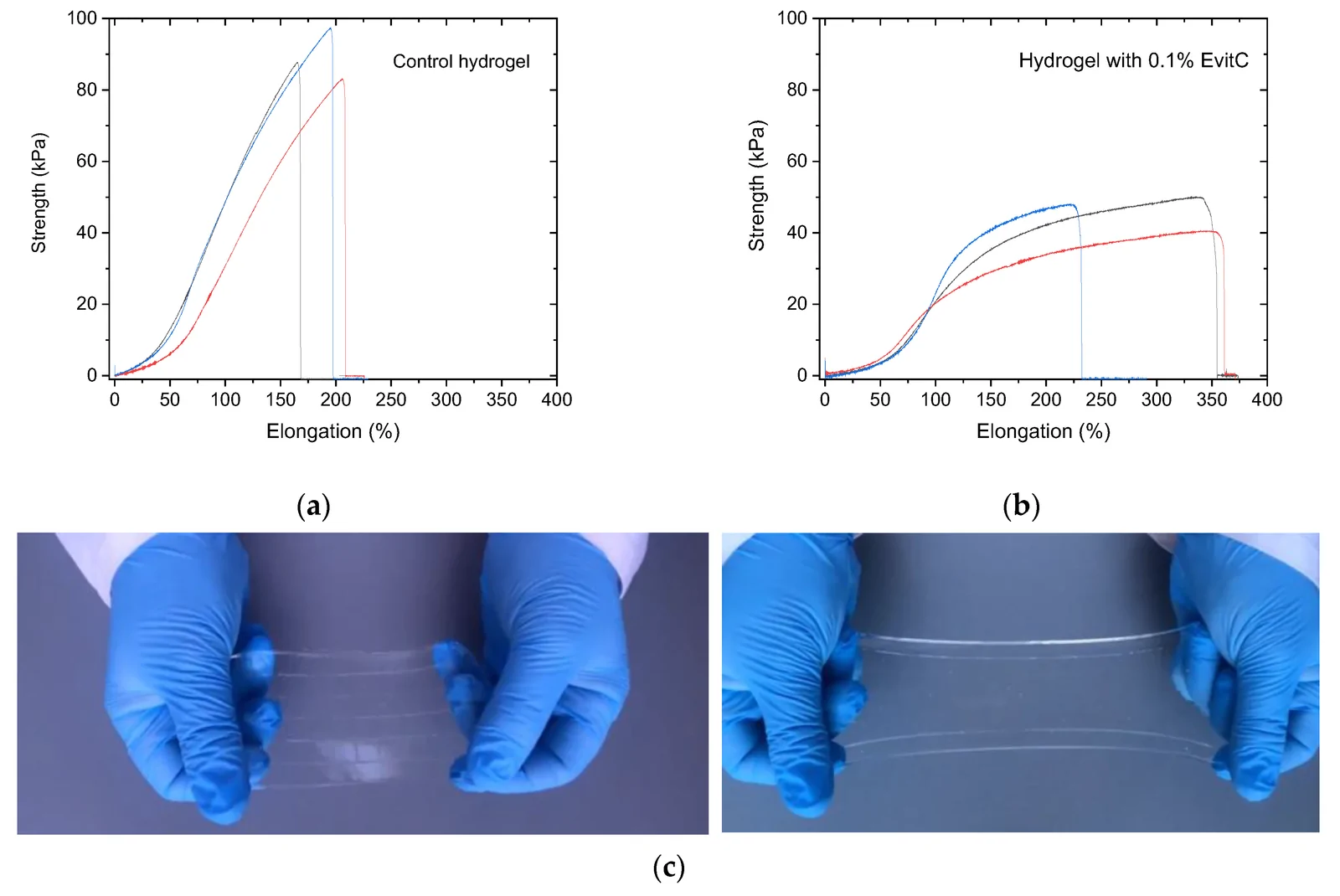
Exemplary stress–elongation dependence of (**a**) reference hydrogels and (**b**) hydrogels with 0.1% EvitC (line colors indicate replicate measurements of the same sample type), and (**c**) real performance of the hydrogel with 0.1% EvitC.

Applications of hydrogels in medicine as wound dressings or in cosmetology for the topical delivery of active substances and skin hydration—the latter is anticipated for hydrogels resulted from the current study—requires appropriate functional properties, such as mechanical strength sufficient for handling and application on the skin. Therefore, tensile properties were evaluated, because they are essential, and more indicative than, for instance, compression strength; Figure 4 presents exemplary stress–elongation dependences and Table 2 collates the tensile parameters.

The maximum strength, elongation at break equaling maximum elongation, and elasticity (Young) modulus are close to the values reported in the literature for similar hydrogel dressings [57]. The presence of the efficient radical scavenger of EvitC in 0.05% and 0.1% affected the mechanical properties of the gel. An apparent trend of reducing maximum strength and increasing the elongation at break is visible, whereas the elasticity modulus remained unchanged (Table 2). The gels become weaker, but the elasticity does not change—it is known that some gels formed by ionizing radiation become brittle at higher doses [58]. A higher content of EvitC resulted in poor mechanical properties of the gels. The chemical crosslinking of PVP was insufficient in upholding the structural integrity of the hydrogel. The physical gel’s, provided by agar, physical interactions are weak and, moreover, the polysaccharide undergoes degradation when irradiated. Both effects resulted in substantial weakening of the gel in comparison to that without the additive. In fact, the gels containing 0.25% of EvitC and 0.5% EvitC, despite some chemical crosslinking, were accomplished, fragmented upon manual handling. In view of the results presented above, hydrogels with 0.1% of EvitC were selected for further investigations.

### 2.3. EvitC Release from Hydrogel

**Figure 5:**
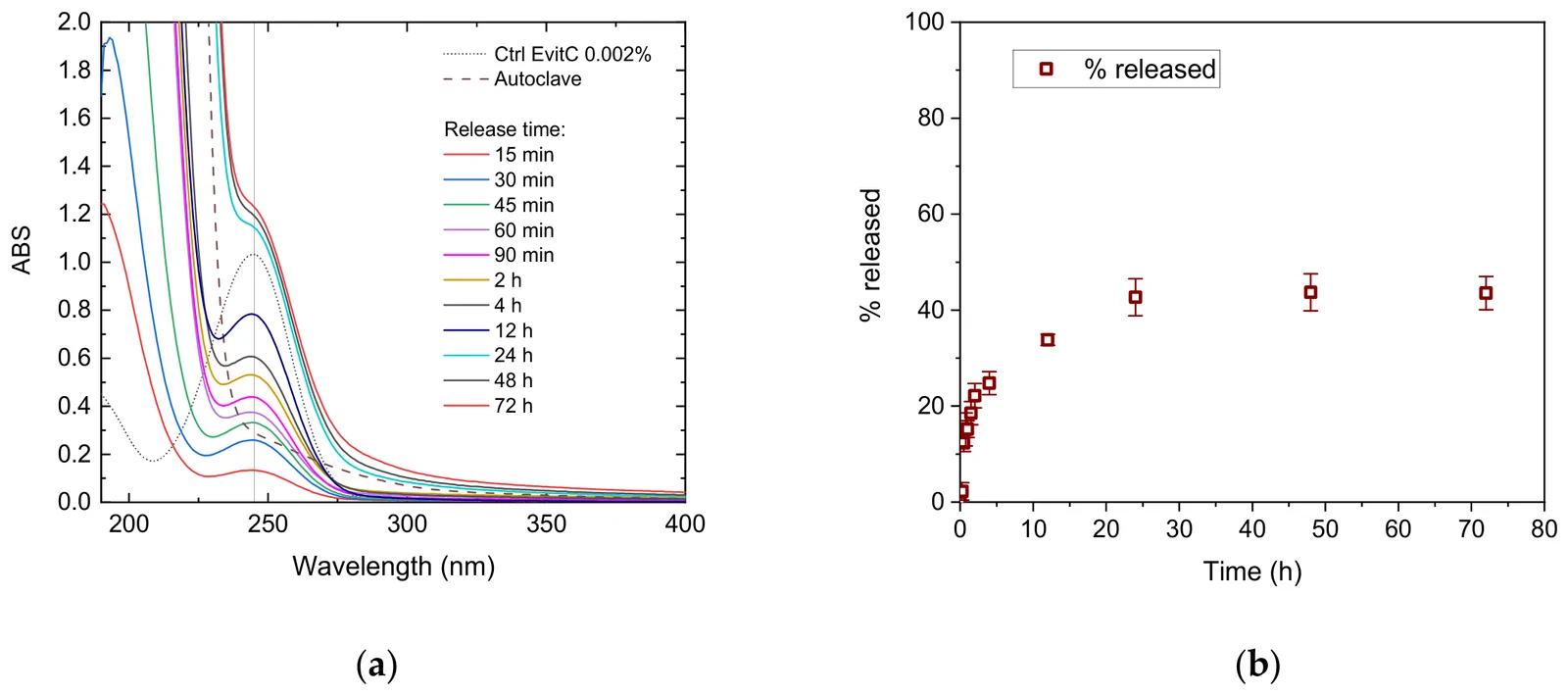
EvitC release from the hydrogel (ca. 2.0–2.5 mm thick) containing 0.1% EvitC as measured with UV-Vis spectrophotometry: (**a**) absorption spectra of the release medium collected up to 72 h, control is a 0.002% EvitC solution in water and the Autoclave represents the absorption of the solution derived after autoclaving the hydrogel with 200 mL water, the vertical line indicates the wavelength of maximum absorption of EvitC; (**b**) release profile as a % of total amount of EvitC present in the hydrogel sample (*n* = 3). Concentration determination was corrected for matrix components’ release (blank—hydrogel without the EvitC) and for reduction in the release medium volume due to sample collection.

In addition to appropriate mechanical properties, one of the most important conditions for a hydrogel to function effectively as a medical device or a cosmetic is the potential for delivering active substances. Active substances are embedded in the hydrogel network during its synthesis—it is a one-pot, green synthesis—and should be released into the skin. The release of EvitC into water from the hydrogel matrix containing 0.1% EvitC was accomplished in order to examine the kinetics of release, and, on the other hand, confirms that EvitC was not entirely degraded by radiation during hydrogel synthesis. Figure 5 presents EvitC release up to 72 h, as measured with UV-Vis spectrophotometry. The amount of released substance is presented as a percentage compared to the total EvitC content in the reaction mixture before irradiation, and thus it does not take into account the part potentially degraded by radiation. About 15% and 43% of EvitC were released from hydrogel in the one-surface contact method, for 60 min and at maximum release time, respectively. This is a relatively small amount, as compared, for instance, to the release profile of active substances of diclofenac or insulin release from cationic hydrogels [59], one of the reasons being the one-sided contact of the EvitC hydrogel with the medium, mimicking the release to the skin (see Section 3.3.3 below).

Release occurs within a very short time from the surface layer. Furthermore, it is assumed that during hydrogel’s topical application—as simulated in this experiment—the active substance diffuses within the hydrogel to the surface. Indeed, this was confirmed through the application of release kinetic models. The data is sufficient for comparing common drug-release models, but one should consider the fitted parameters exploratory rather than definitive (Table 3). The release data were best described by the Weibull model: R^2^ = 0.974 and 0.966, the latter under assumption that the maximum release is less than 50% of the substance amount present in the initial mixture (see the Discussion below). This, combined with β = 0.472, indicates predominantly Fickian-diffusion-driven release. The Korsmeyer–Peppas model yielded a release exponent of n = 0.765, calculated for the data up to 4 h, which is roughly 60% of the maximum released (R^2^ = 0.732), and is somewhat distinct from the theoretical value for Fickian diffusion—approximately 0.45 for slabs, but higher for changing diffusion geometries, such as due to polymer chains relaxation and swelling, and due to diffusion of the active substance by the water-filled regions of the hydrogel [60,61]. These results indicate that drug transport from the hydrogel is predominantly governed by diffusion, however anomalous in its nature. Moreover, the Weibull model describes the entire time curve and is primarily empirical, whereas Korsmeyer–Peppas evaluates the initial release regime. This may indicate that polymer swelling/relaxation contributes particularly during the early phase, after which diffusion becomes increasingly dominant. The somewhat poorer fit of the Higuchi model compared with Weibull reflects also hydrogel’s swelling effects and one-sided exposure geometry. The asymmetric geometry of the system, in which only one surface of the hydrogel was exposed to the release medium, is consistent with diffusion-controlled release through a partially restricted matrix. Apparently, this is neither zero-order release nor erosion-controlled release, as demonstrated by the poor fitting to the Hixson–Crowell model. As the active substance passes into the skin and is gradually absorbed upon real application conditions, a concentration gradient mechanism starts to operate here, and the driving force is the diffusion within the hydrogel network. It should be noted that distilled water was used as a simplified release medium; therefore, the obtained release profile may not fully reflect EvitC release under physiological conditions. Further studies using physiologically relevant media, such as PBS or simulated wound fluid, are required to evaluate release behavior under conditions more representative of topical application. The presented results demonstrate the release of the active component from the carrier and should be regarded as the maximum possible rate of delivery, and pharmaceutical availability to the skin will be somewhat lower. This may be further simulated ex vivo with skin models or examined in real in vivo experiments [26].

The protective effect of the components of the reaction mixture, i.e., polymers, towards the active substance is confirmed. It is evident when comparing the substantial decomposition of EvitC irradiated in water (Figure 2; 2 kGy decomposes 70% of the substance) with the fact that it is present in the hydrogel matrix after synthesis and is released in a larger amount than if it was decomposed to the same extent as in water. Such protective effect is often observed, as the energy of radiation is absorbed by the solutes with respect to their mass fraction, through reactions with water radiolysis transient products [62]. The probability of a reaction of water-derived reactive species with a particular solute depends also on the respective rate constants. For instance, hydroxyl radical reacts with the polymeric components slower, but their concentration is 100-fold higher than the concentration of the active substance. A rough estimate can be made on the extent of the protective effect of the hydrogel components on EvitC. The fraction of EvitC degraded by radiation-induced reactions during hydrogel synthesis or covalently coupled with polymers by radical recombination can also be estimated by kinetic simulations (see Supplementary Materials). Based on component concentrations and known rate constants or typical ranges thereof for the given reaction type, the upper limit of EvitC loss was estimated at 58%, indicating that at least 42% of the EvitC should remain intact and free within the gel matrix structure. This value shows good agreement with the total fraction of the initial EvitC eluted from the gel matrix at the longest studied time of 72 h. It is necessary to stress that partial decomposition of the active component is the main drawback of the one-pot green synthesis of hydrogels by radiation. Yet, the data and presented estimation demonstrated that EvitC is present in the hydrogel after synthesis, and is available for release in foreseen applications; for instance, as a cosmetic moisturizing and regeneration hydrogel mask.

### 2.4. Testing of Drying

**Figure 6:**
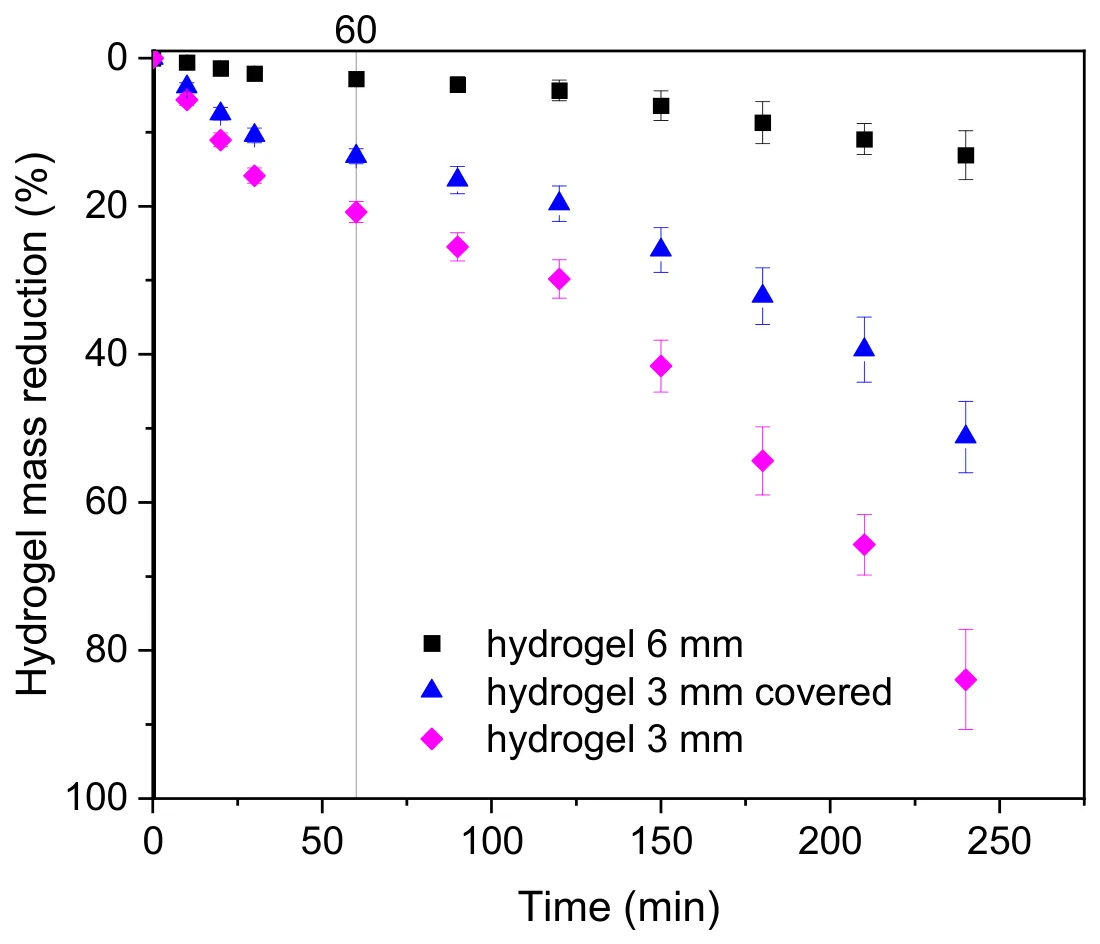
Mass loss of hydrogel with 0.1% EvitC due to drying on the surface of a hot plate at 37 °C. Hydrogel of 3 mm thickness, covered with nonwoven fabrics, and hydrogel of 6 mm thickness. Average values and standard deviation, *n* = 3. The vertical line indicates typical time of hydrogel application.

It is known that hydrogels in medical applications may be used as wound dressings, particularly for burn wounds, because hydrogel reduces the temperature through absorbing excess heat from the injured site. It is due to continuous water evaporation that the surface under a hydrogel dressing is cooler by several degrees Celsius in comparison to the skin not covered with the hydrogel [63]. Figure 6 presents the drying kinetics (mass loss) of hydrogel containing 0.1% EvitC in three variants—regular hydrogel of 3 mm thickness, the same but covered with dense nonwoven fabrics, and hydrogel of 6 mm, i.e., doubled thickness. The duration of maintaining hydrogels’ wet state—providing a moisturizing function, important for cosmetic applications—can be controlled by the thickness of the hydrogel and the supporting covering. Apparently, the cooling effect will be reduced in the covered hydrogel due to slower water evaporation. Nevertheless, this option may be convenient for prolonged, even 4–5 h, usage, providing moisturizing and release effects. It should be noted that practical use on the skin will result in slower evaporation due to lowering the skin temperature and, of significant importance, EvitC will penetrate moisturized, relaxed skin more efficiently, enhancing its biological effect.

### 2.5. Long-Term Stability

It is of practical importance that the hydrogel maintains its properties and activity for a prolonged time after manufacturing, until the point of use. Therefore, one-year-storage of the hydrogels was simulated through the application of so-called accelerated conditions—fifteen weeks of storage at 40 °C. Table 4 provides the data of selected parameters’ change after the simulated one-year storage. Only minor changes in GF, EDS and tensile properties may indicate that the hydrogel, in general, maintained its essential parameters. However, one may speculate that slow degradation, or hydrolysis of the network, proceeds, even though hydrogel products of a similar nature are available in the market with two years of shelf-life. Looser network after one year aging can be confirmed through the faster release of EvitC. Nevertheless, the active component did not decompose and is available in the hydrogel. Observations on the photostability of vitC in gels, as measured by UV-Vis spectrophotometry, have been reported, indicating the crucial effects from both the nature of the gel and the type of vitC derivative [64]. As visible radiation was used in those experiments, the factor of gel transparency was critical. Our experiment confirmed that the composition and applied radiation-based technology resulted in a stable product. What is also worth mentioning is that the hydrogels were not contaminated by microorganisms, as there were no indications of spoilage, i.e., visible mold or bacterial colonies. Apparently, during the radiation synthesis, sterilization was accomplished (see Supplementary Materials for further explanation).

### 2.6. Biological Evaluation of Ethyl Ascorbic Acid

To verify the biological safety of the EvitC and select its concentrations for incorporation into the hydrogel formulation, its effect on human dermal fibroblast viability was evaluated using the XTT assay. Cell viability was determined after 24 and 48 h of incubation.

**Figure 7:**
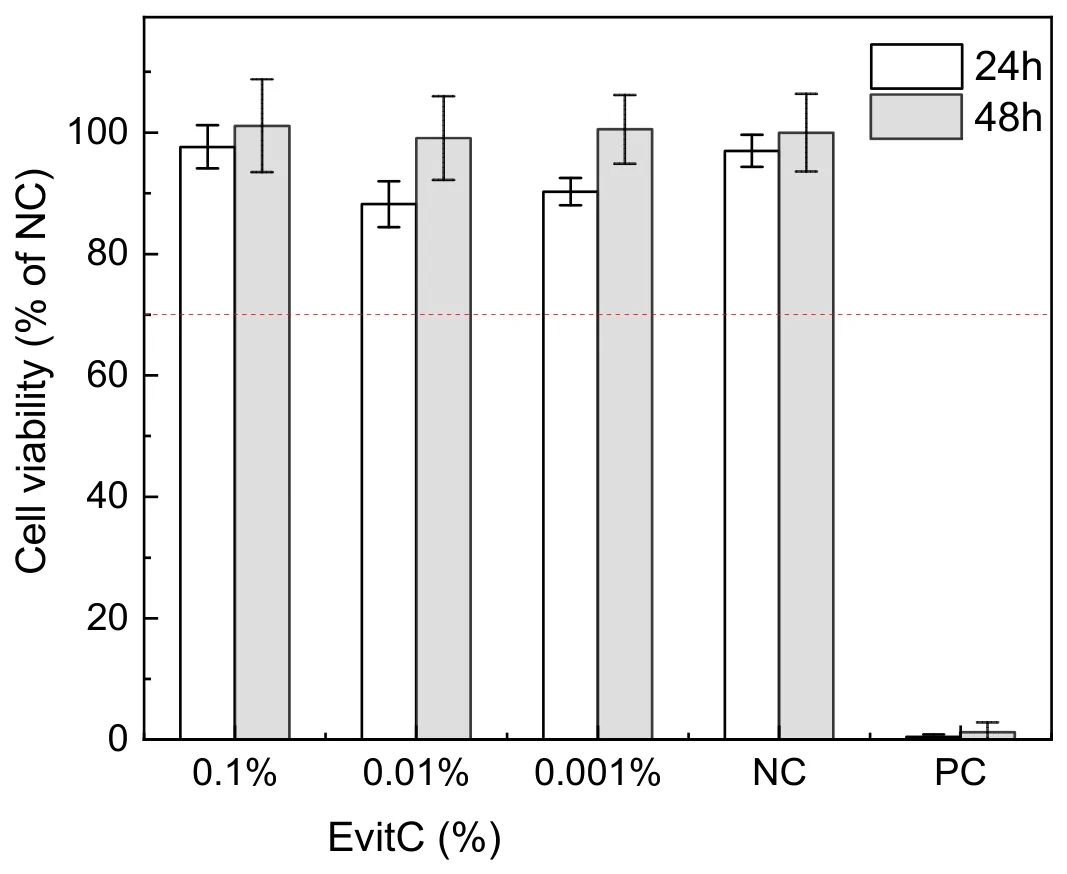
XTT—viability of hBF after 24 and 48 h exposure to EvitC solutions of 0.1, 0.01 and 0.001% concentrations, relative to the negative control (NC)—medium with cells; PC is the positive control (cells with Triton X-100). The red horizontal line denotes the 70% cytotoxicity threshold.

As shown in Figure 7, none of the tested EvitC concentrations, i.e., 0.1, 0.01 and 0.001%, reduced cell viability below the 70% threshold specified in ISO 10993-5 [65], indicating the absence of cytotoxic effects. After 24 h of incubation, a slight decrease in viability was observed for some samples when compared with the NC, whereas the 0.1% concentration maintained viability at a level comparable to the control. After 48 h, cell viability remained comparable to that of the NC for all tested concentrations, indicating that the initial reduction in metabolic activity was transient. These findings confirm the biological safety of EvitC in concentrations of up to 0.1%. These observations are in agreement with previous studies reporting the good cytocompatibility of 3-O-ethyl-L-ascorbic acid-containing serum toward skin cells, supporting the suitability of this stabilized vitC derivative for topical applications [66].

**Figure 8:**
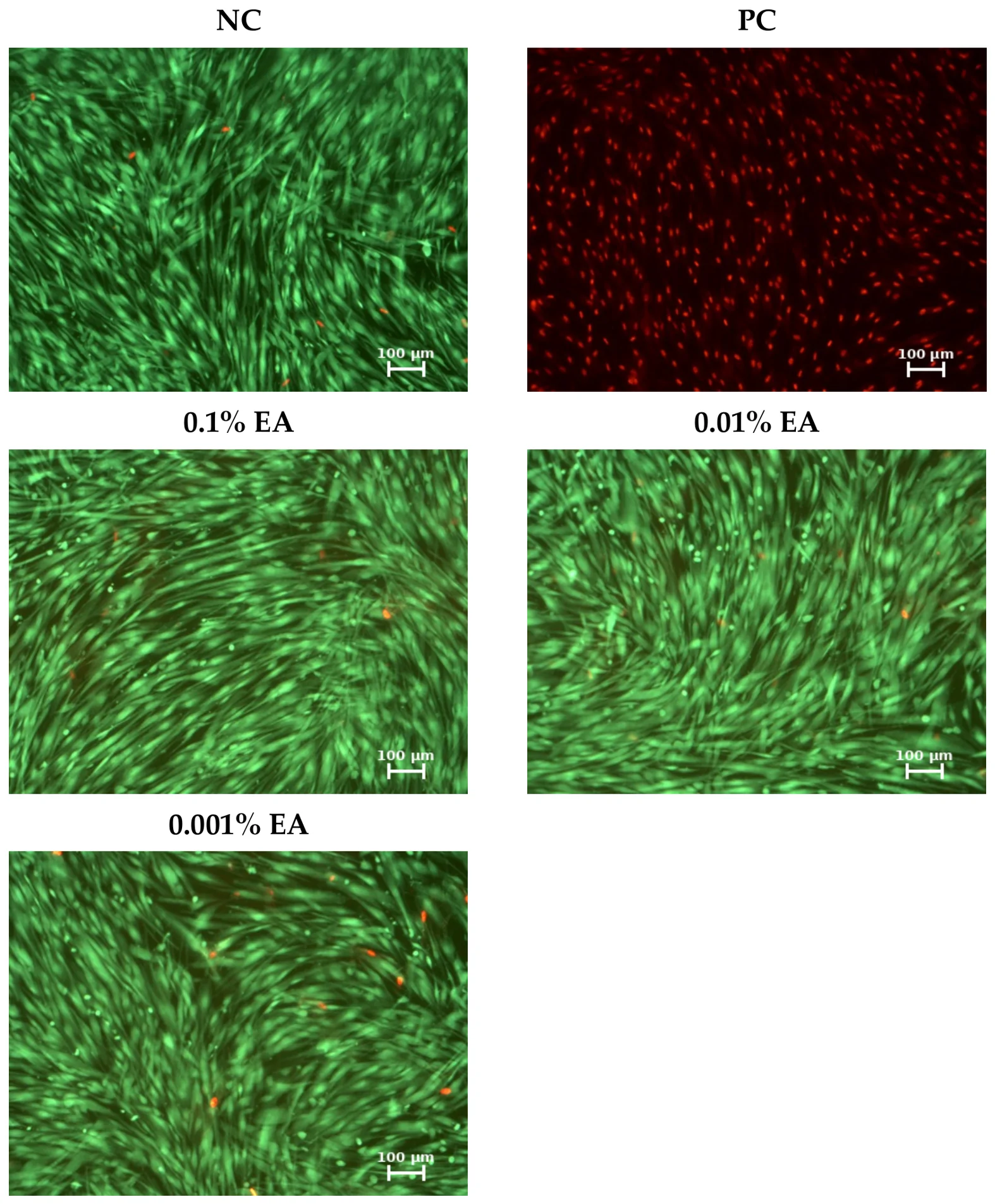
Representative Live/Dead fluorescence images of hFB (at ×100 magnification) after 48 h exposure to EvitC solutions. Live cells are shown in green (calcein-AM) and dead cells in red (EthD-1). NC is the negative control—medium with cells—and PC is the positive control (cells with Triton X-100).

The Live/Dead assay was performed to further evaluate the effect of EvitC on fibroblast viability after 48 h of incubation. Fluorescence images in Figure 8 confirm the XTT results—a dense layer of viable fibroblasts with only a few EthD-1-positive cells at all tested EvitC concentrations. Moreover, fibroblasts retain their characteristic elongated, spindle-shaped morphology, comparable to that observed in the NC. In contrast, the PC exhibits a predominance of dead cells, confirming the effectiveness of the staining procedure. These observations further demonstrate that EvitC concentrations of up to 0.1% did not adversely affect fibroblast viability or morphology and justify the incorporation of EvitC into the hydrogel formulation, at concentrations selected based on physicochemical evaluation.

### 2.7. Biological Evaluation of Hydrogel Containing EvitC

The biological effects of extracts of hydrogel containing 0.1% EvitC were evaluated using complementary in vitro assays to assess their cytocompatibility and effects on ECM-associated collagen and elastin deposition in human dermal fibroblasts. hFB viability was determined using the XTT assay and qualitatively confirmed by Live/Dead staining, whereas ECM-associated collagen and elastin deposition were assessed using the Col-F assay. Hydrogel extracts were prepared through 2 or 12 h extraction (Col-F for 2 h) and were tested both undiluted (100%) and after 1:1 dilution with complete culture medium (50%).

**Figure 9:**
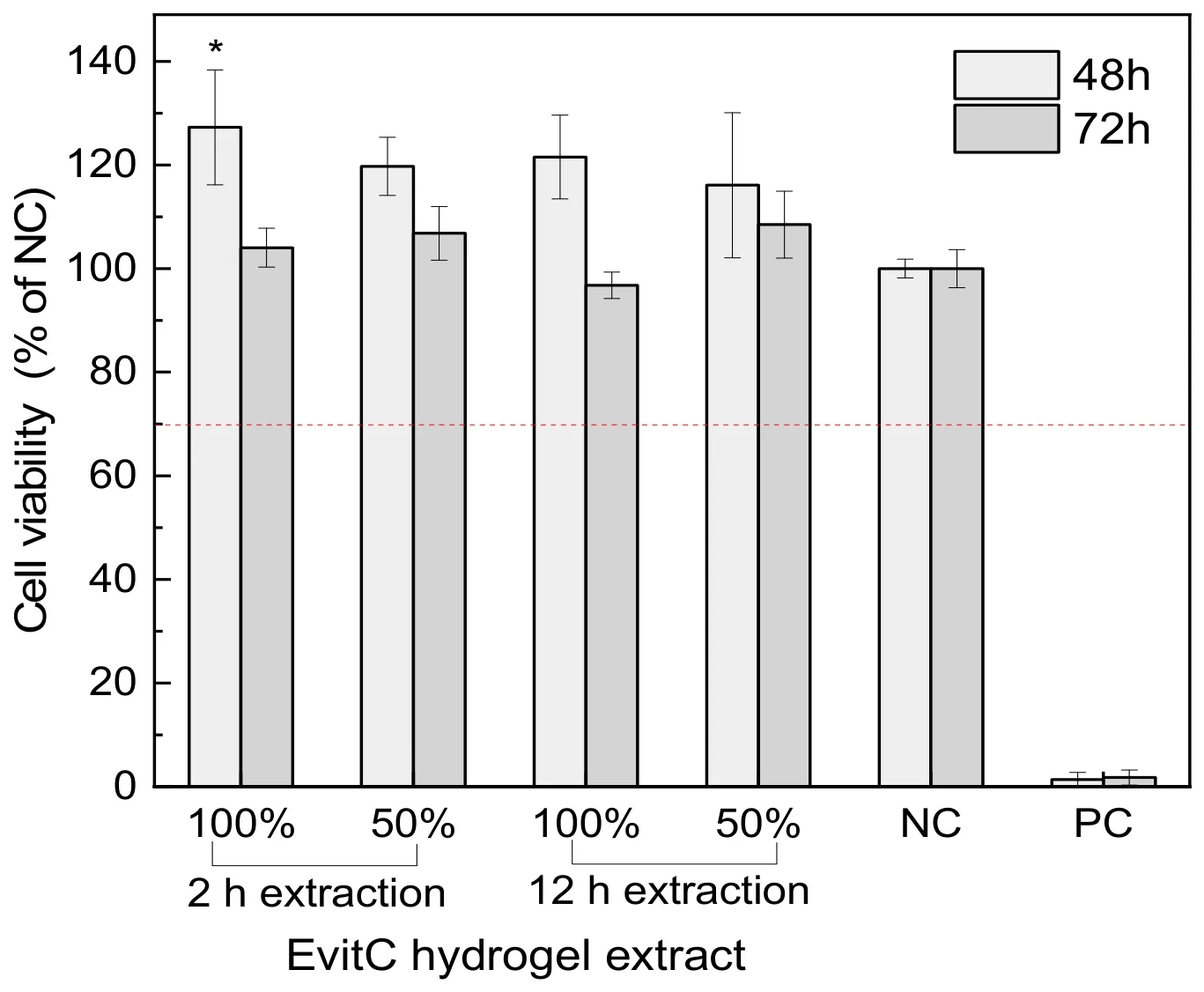
XTT—viability of hFB exposed to extracts from hydrogels containing 0.1% EvitC after 48 and 72 h of exposure. Hydrogel extracts were prepared through 2 or 12 h extraction and tested either undiluted (100%) or after 1:1 dilution with complete culture medium (50%). NC is the negative control—medium with cells—and PC is the positive control (cells with Triton X-100). Data are presented as mean ± SD from three independent experiments (*n* = 3), with six technical replicates per experimental condition in each experiment. Technical replicates were averaged within each independent experiment before statistical analysis. At each incubation time, individual hydrogel extract groups were compared with the corresponding NC using Dunnett’s multiple-comparison test. * indicates a statistically significant difference compared with NC (*p* < 0.05). The red horizontal line denotes the 70% cytotoxicity threshold.

Cell viability after 48 and 72 h of incubation are presented in Figure 9. Viability of hFB incubated with all hydrogel extracts was above the 70% threshold specified in ISO 10993-5, confirming the absence of cytotoxic effects regardless of extraction time and extract concentration. Moreover, the mean metabolic activity of cells exposed to the hydrogel extracts remained at or above the level of the NC under all experimental conditions, except for the 12 h undiluted extract after 72 h, for which the mean value remained close to the NC. After 48 h, mean metabolic activity ranged from approximately 116 to 127% of the NC. Dunnett’s multiple-comparison test revealed a statistically significant increase in metabolic activity for cells exposed to the undiluted extract prepared by 2 h extraction (2 h-100%) compared with the NC (*p* = 0.026). No statistically significant differences from the NC were observed for the 2 h-50%, 12 h-100%, or 12 h-50% extracts (*p* > 0.05). After 72 h, metabolic activity ranged from approximately 97 to 109% of the NC, and none of the hydrogel extract groups differed significantly from the NC (*p* > 0.05). Thus, the increase observed for the 2 h-100% extract after 48 h was not maintained after prolonged exposure, with metabolic activity approaching the NC level after 72 h. Importantly, all viability values remained well above the 70% cytotoxicity threshold, indicating that none of the tested hydrogel extracts exerted a cytotoxic effect. The comparable biological response observed for extracts prepared through 2 and 12 h of extraction further supports the cytocompatibility of the released compounds. These findings indicate that incorporation of 0.1% EvitC into the biocompatible hydrogel matrix does not induce cytotoxicity and supports its application as a hydrogel-based delivery system for this active compound.

**Figure 10:**
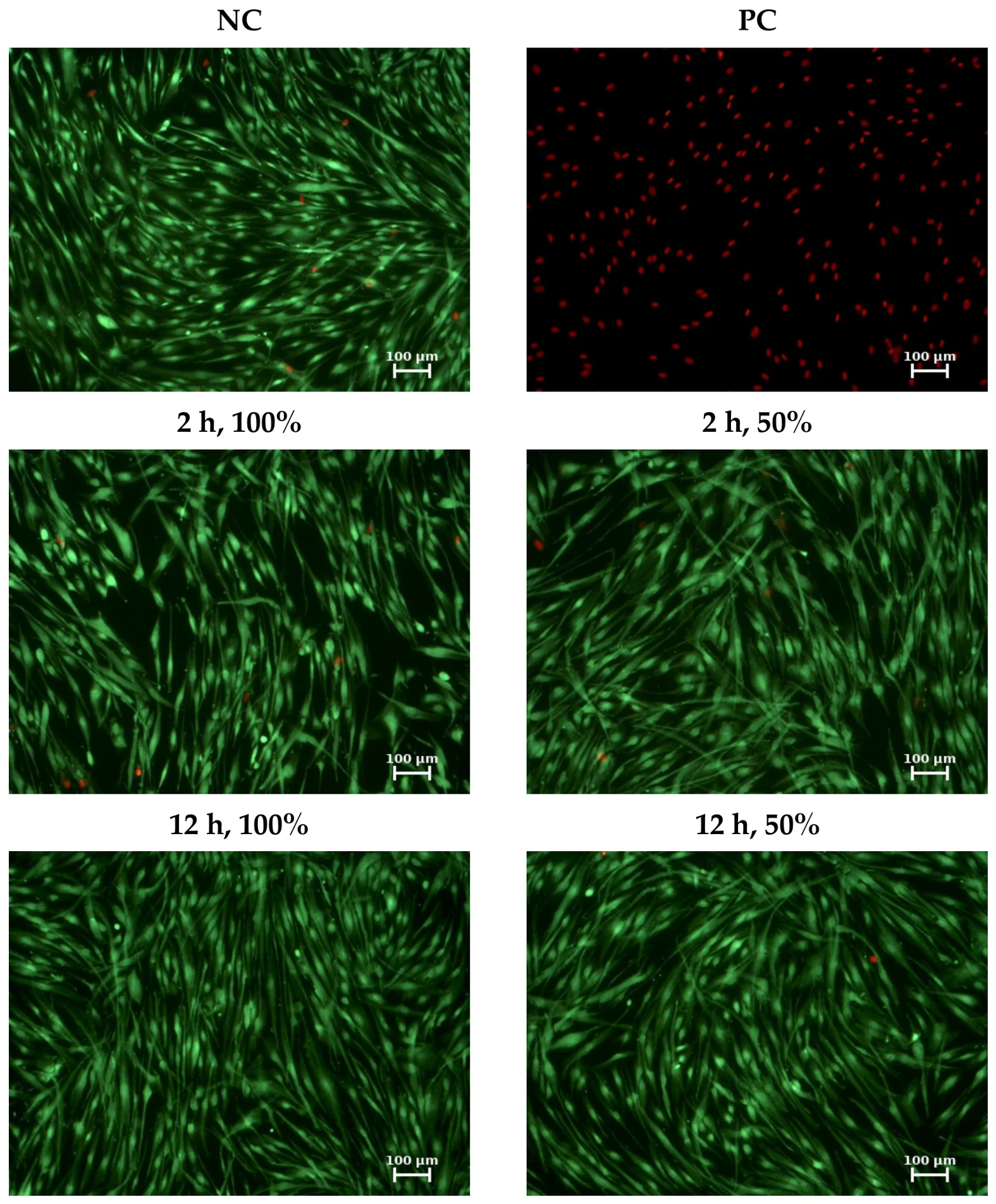
Representative Live/Dead fluorescence images of hFB (at ×100 magnification) following their 72 h exposure to extracts from hydrogels containing 0.1% EvitC. Hydrogel extracts were prepared through 2 or 12 h of extraction and tested either undiluted (100%) or after 1:1 dilution with complete culture medium (50%). Live cells are shown in green (calcein-AM), whereas dead cells are shown in red (EthD-1). NC is the negative control—medium with cells—and PC is the positive control (cells with Triton X-100).

To qualitatively confirm the XTT findings, fibroblast viability and morphology were evaluated using the Live/Dead assay after 72 h of incubation. The fluorescence images in Figure 10 show a dense population of viable fibroblasts with only a few EthD-1-positive cells in all experimental groups. hFB exposed to both undiluted and diluted hydrogel extracts retained their characteristic elongated, spindle-shaped morphology and formed a confluent monolayer comparable to that of the NC. No qualitative differences in cell morphology or the distribution of viable and dead cells were observed between extracts prepared after 2 or 12 h of extraction. In contrast, the positive control exhibits predominantly EthD-1-positive cells, confirming the validity of the staining procedure. These observations are consistent with the XTT results and further confirm the sound cytocompatibility of the developed EvitC-containing hydrogel. Similar unaltered cytocompatibility has been reported for 3-O-ethyl-L-ascorbic acid-containing topical serum, although the present study further demonstrates that EvitC retains its biological safety following radiation crosslinking and release from the hydrogel matrix [66].

**Figure 11:**
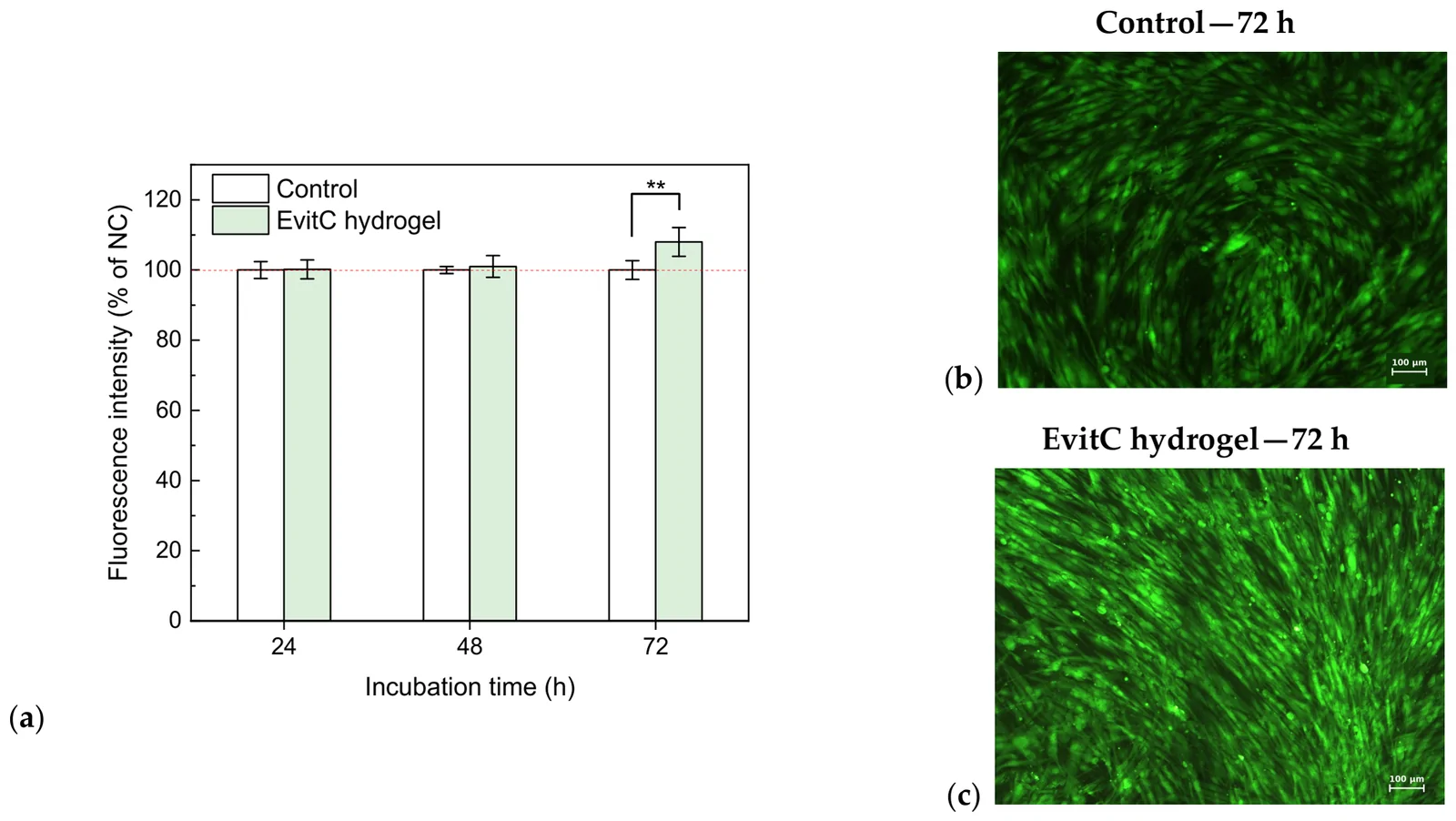
Relative Col-F fluorescence intensity (**a**) and representative fluorescence images of hFB (**b**,**c**) following exposure to extracts prepared through 2 h extraction from hydrogels containing 0.1% EvitC. Fluorescence intensity was measured after 24, 48 and 72 h of incubation and normalized to untreated control cells (100%). Representative Col-F fluorescence images of untreated control cells and cells exposed to hydrogel with EvitC extracts after 72 h are shown on the right (×100 magnification). Green fluorescence indicates collagen and elastin deposition within the extracellular matrix. Data are presented as mean ± SD (*n* = 3). Statistical significance between the EvitC hydrogel extract and the corresponding control was assessed using a paired two-tailed Student’s *t*-test; ** *p* < 0.01. The red horizontal line serves as a visual guide indicating the control level.

Following confirmation of the cytocompatibility of the hydrogels with EvitC, their effect on extracellular matrix production was evaluated using the Col-F assay. hFB were exposed to hydrogel extracts, prepared through 2 h extraction, for 24, 48 and 72 h. As shown in Figure 11, no noticeable differences in Col-F fluorescence intensity were observed between the control and cells exposed to EvitC hydrogel after 24 or 48 h of incubation. After 72 h, cells exposed to extracts from hydrogels containing EvitC showed the highest fluorescence intensity, corresponding to approximately 108% of the control. Although the increase was moderate, it was statistically significant compared with the corresponding control (paired two-tailed Student’s *t*-test, *p* = 0.007). Representative fluorescence images obtained after 72 h are consistent with the quantitative measurements, showing a more intense Col-F fluorescence signal in fibroblasts exposed to hydrogel with EvitC extracts than in control cells, while preserving their characteristic elongated, spindle-shaped morphology. Previous studies on closely related radiation-crosslinked PVP/PEG/agar hydrogels demonstrated good cytocompatibility, including extract-based testing, and no significant increase in fibroblast growth compared with control conditions [67,68]. Vitamin C is a cofactor for prolyl and lysyl hydroxylases involved in collagen biosynthesis and is known to promote collagen production by dermal fibroblasts, thereby supporting extracellular matrix formation [2,7]. Similarly, Zerbinati et al. reported enhanced collagen production by dermal fibroblasts following treatment with 3-O-ethyl-L-ascorbic acid-containing serum while maintaining excellent cytocompatibility [66]. Therefore, the tendency toward increased Col-F fluorescence observed after 72 h is consistent with the known biological activity of EvitC and suggests that EvitC released from the hydrogel may contribute to extracellular matrix production. However, since a blank hydrogel extract was not included in the present experimental design, a contribution from the hydrogel matrix or its extractables cannot be completely excluded. As the Col-F assay provides an indirect assessment of ECM-associated collagen and elastin deposition, further biochemical or molecular studies are required to confirm enhanced ECM synthesis.

Based on the obtained results from biological examination with human dermal fibroblasts, the developed 3-O-ethyl-L-ascorbic acid-containing hydrogel appears to be a cytocompatible delivery platform with the potential to support fibroblast function and extracellular matrix production, particularly for cosmetic applications. Although the beneficial biological properties of EvitC have previously been described in other forms of delivery, the present study suggests that its biological activity may be retained following incorporation into the hydrogel and its subsequent release from the polymer network. It should also be noted that the present biological evaluation was limited to human dermal fibroblasts. Further studies using additional skin-relevant cell types or more complex co-culture models would provide a more comprehensive assessment of the biological response to the developed hydrogel.

Compared with other approaches used to improve the stability and topical delivery of vitamin C and its derivatives, including liposomal systems, nanoemulsions, microneedle-based formulations, and hydrogel carriers [69], the radiation-crosslinked hydrogel developed in this study represents a relatively simple polymeric platform combining high water content with the sustained release of EvitC. Unlike multicomponent nanocarrier systems, the proposed formulation does not require the preparation of dispersed nanoscale carriers, while radiation processing enables crosslinking and sterilization of the hydrogel in a single technological step. From a manufacturing perspective, radiation processing is compatible with controlled large-scale production, although detailed assessment of production costs and process scalability was beyond the scope of this work. The demonstrated stability of the formulation during storage further supports its practical potential. Nevertheless, additional studies addressing skin penetration, more complex biological models, and scale-up considerations will be required before translation into a final topical product.

## 3. Materials and Methods

### 3.1. Materials

Poly(N-vinylpyrrolidone) (PVP, K-90), poly(ethylene glycol) (PEG-400) and agar were purchased from BASF (Ludwigshafen, Germany), Sigma-Aldrich (St. Louis, MO, USA), and BD (Becton, Dickinson and Company, Franklin Lakes, NJ, USA), respectively. Four types of vitamin C, i.e., L-ascorbic acid, ascorbyl glucoside, 3-O-ethyl-L-ascorbic acid (termed ethyl ascorbic acid or EvitC), and sodium L-ascorbyl-2-phosphate (Na-ascorbyl phosphate) (Selco Wirkstoffe Vertriebs GmbH, Wald-Michelbach, Germany), were used as received.

### 3.2. Hydrogel Manufacturing and Irradiation

The hydrogel was obtained via the method developed for the synthesis of hydrogel dressings for medicine, which employs ionizing radiation to form a three-dimensional polymer network in aqueous systems [70,71]. The reaction mixture of PVP, PEG, and optionally EvitC, was prepared by dissolving the components in water at elevated temperatures (over 50 °C) and homogenized by continuous mixing with agar dissolved in boiling water. The resulting solution, containing 6% PVP, 2% PEG, 1% agar, and optionally EvitC, was cast into molds and left at R.T. for solidification due to the physical interactions provided by the polysaccharide. The samples were sealed in polymer packaging and irradiated. Crosslinking of the synthetic polymer was achieved by irradiation with a dose of 25 kGy, using the electron beam generated by a 6 MeV linear accelerator (ELU-6). The irradiation dose was determined by calorimetric dosimetry. The high dose rate of ca. 5 kGy/min is advantageous in providing high temporal concentration of radicals in the system crucial for the biradical reactions of recombination, forming crosslinks between macromolecules. There is at least a one-order-of-magnitude-higher dose rate that can be obtained in powerful gamma installations. It is anticipated that the resulting hydrogels were sterile. Aqueous solutions of EvitC were irradiated with the same machine, at doses up to 2 kGy.

### 3.3. Physicochemical Examination

#### 3.3.1. Degree of Swelling and Gel Fraction

The swelling degree at equilibrium (EDS) is determined as the ratio of the mass of the swollen hydrogel (after water absorption) to the mass of the hydrogel after synthesis (the mass of all components, including water, are taken into account). The tests were performed on gel disks with a constant diameter of 23 mm and a thickness of ca. 3 mm. The swelling of the hydrogels was determined at room temperature by systematic weighing of the samples until three consecutive results did not differ by more than ±5% (up to 7 days)—the gel reached the equilibrium (EDS). The swelling degree of the hydrogels was calculated using the formula:

(1) $$EDS=\frac{m_{s}-m_{0}}{m_{0}}100\%$$

where EDS—degree of swelling at equilibrium, m_0_—mass of the initial hydrogel after synthesis [g], m_s_—mass of the hydrogel at equilibrium swelling [g].

Gel fraction (GF) refers to the amount of crosslinked polymer in the matrix and is determined as the ratio of the dry mass of the gel after sol removal (extraction) to the mass of all hydrogel components other than water (and possibly the bioactive substance) present in the gel after synthesis. After reaching equilibrium swelling, the hydrogels were autoclaved (121 °C, 15 min) to completely remove the sol fraction. In the next step, the gel fractions were dried to constant mass in an oven. Knowing the initial mass of the polymer in the sample before irradiation, the gel fraction content was calculated using the equation:

(2) $$GF=\frac{m_{g}}{m_{p}}100\%$$

where GF is the gel fraction and m_p_—mass of polymers in the sample [g], m_g_—mass of dried gel [g]. The EDS and GF were evaluated in triplicate and the average was determined.

#### 3.3.2. Tensile Mechanical Tests

For tensile strength testing, hydrogels were cut out to the standard ‘dumbbell’ shape with a length/width of the gauge section of 50 mm/4 mm. The Z2.5 (Zwick Roell, Ulm, Germany) tensile instrument was used with a 100 N loading head, moving at the speed of 50 mm/min. Knowing the sample cross-cut area and based on stress and strain measurements, maximum stress and elongation were determined. Using the typical tensile stress curve of soft materials, such as polymer hydrogels, the elasticity (Young) modulus E, was calculated based on the slope of the stress–strain curve in the steepest strain range. Tensile tests were conducted in triplicate and the average was determined.

#### 3.3.3. EvitC Release Studies and UV-Vis Spectrophotometry

The tests of EvitC release were conducted using hydrogels disks of 23 mm diameter. The disks were placed on a net floating on the surface of 50 or 80 mL of water of temperature 37 °C. The hydrogel bottom was immersed in water for not more than 1 mm; therefore, only one surface was contributing to EvitC release, mimicking topical applications. The 3 mL aliquots were collected at fixed time intervals for up to 72 h. The samples were analyzed by UV-Vis spectrophotometry (Shimadzu UV2600, Kyoto, Japan) in a 10 mm optical length cell, at a spectral range of 190–400 nm against the water reference. Release experiments were conducted in triplicate and the average was determined. The same spectrophotometer was used for initial screening of experiments of vitamin C’s thermal and radiation stability.

#### 3.3.4. Hydrogel Drying Analysis

The hydrogels, in their so-called relaxed state, i.e., after synthesis, in 23 mm diameter disks were weighed and placed on foil on a heating plate at 37 °C, simulating operating conditions—gels were uncovered or covered with a fabrics imitating a bandage. Drying occurred through water evaporation from the upper surface of the hydrogel. The gels were weighed at specified intervals. The percentage mass loss was determined using the following formula:

(3) $$\mathrm{Mass}\mathrm{loss}=\frac{m_{0}-m_{t}}{m_{0}}100\%$$

where m_0_—mass of the initial hydrogel after synthesis [g], m_t_—mass of the hydrogel after time t [g].

#### 3.3.5. Long-Term Stability Testing in Accelerated Conditions

The generally accepted method for testing product stability, or shelf-life, is demonstrated in the ASTM F1980 guidance [72]. The temperature used for accelerated aging considered the elevated-temperature stability of the components of the hydrogels being tested, primarily the active ingredient. Accordingly, 40 °C was chosen, because all components of the composition do not decompose at this temperature. Hydrogels were heat-sealed in moisture-impermeable foil bags and stored in the laboratory drier. Fifteen weeks at 40 °C simulates natural storage conditions for 12 months at room temperature (22 °C assumed). Afterwards, hydrogel samples were examined with selected tests.

### 3.4. In Vitro Biological Evaluation

#### 3.4.1. Cell Culture

Human dermal fibroblasts (hFB; ATCC^®^, PCS-201-012) were cultured in Fibroblast Basal Medium (ATCC^®^, PCS-201-030) supplemented with Fibroblast Growth Kit Low Serum (ATCC^®^, PCS-201-041) all purchased from ATCC (Manassas, VA, USA) according to the manufacturer’s recommendations. Cells were maintained at 37 °C in a humidified atmosphere containing 5% CO_2_ and passaged upon reaching approximately 80% confluence. For biological assays, fibroblasts were seeded in 96-well culture plates at a density of 6 × 10^3^ cells/well in 100 µL of complete culture medium and allowed to attach for 24 h before treatment.

#### 3.4.2. Preparation of EvitC Solutions and Hydrogel Extracts for Biological Evaluation

Ethyl ascorbic acid (EvitC) was dissolved in complete culture medium to obtain working solutions at concentrations of 0.1, 0.01 and 0.001% and used immediately after preparation. Hydrogel extracts were prepared according to ISO 10993-12 [73] by incubating hydrogel samples in complete culture medium at an extraction ratio of 0.2 g/mL at 37 °C for 2 or 12 h, depending on the experimental design. An additional volume of culture medium was added during extract preparation to compensate for the medium uptake by the hydrogel. Hydrogel extracts prepared after both 2 and 12 h of extraction were used for the XTT and Live/Dead assays, whereas only 2 h extracts were used for the Col-F assay. Freshly prepared extracts were used for biological evaluation, either undiluted (100%) or after 1:1 dilution with complete culture medium (50%), depending on the assay.

#### 3.4.3. Cytocompatibility—XTT Assay

Cell metabolic activity was evaluated using the XTT Cell Proliferation Assay Kit (Biological Industries, Cromwell, CT, USA) according to the manufacturer’s protocol. hFB were seeded into 96-well plates at a density of 6 × 10^3^ cells/well (100 µL) and cultured for 24 h. The culture medium was subsequently replaced with 100 µL of EvitC solutions or hydrogel extracts prepared as described above. Cells cultured in complete medium were used as the negative control (NC), whereas cells treated with Triton X-100 (Sigma-Aldrich (St. Louis, MO, USA) were the positive control (PC). For EvitC solutions, the metabolic activity of cells was determined after 24 and 48 h, whereas cells’ exposure to hydrogel extracts were evaluated after 48 and 72 h of incubation.

Following incubation, XTT reagent was added to cells according to the manufacturer’s protocol and absorbance was measured at 450 nm with a reference wavelength of 630 nm using a Synergy HT microplate reader (BioTek Instruments, Winooski, VT, USA). Cell metabolic activity was expressed as a percentage of the negative control, which was set as 100%. Values ≥ 70% of the NC were considered non-cytotoxic in accordance with ISO 10993-5. Each experimental condition was evaluated in 6 technical replicates in three independent experiments (*n* = 3).

#### 3.4.4. Cell Viability—Live/Dead Assay

Cell viability was assessed using the LIVE/DEAD^®^ Viability/Cytotoxicity Kit (Invitrogen, Carlsbad, CA, USA). hFB were cultured under the conditions described above and exposed to either EvitC solutions or hydrogel extracts. Following incubation (48 h for EvitC solutions and 72 h for hydrogel extracts), the culture medium was removed, and the cells were washed twice with phosphate-buffered saline (PBS). The cells were then incubated with 1.0 μM ethidium homodimer-1 (EthD-1) and 0.375 μM calcein-AM in PBS for 30 min at 37 °C. After staining, the cells were washed with PBS and examined using an inverted fluorescence microscope equipped with a CCD camera (Axio Observer Z1, Carl Zeiss, Jena, Germany). Calcein-AM-positive viable cells were visualized using a 09AF488 filter, whereas EthD-1-positive dead cells were visualized using a 20 Rhod filter. Representative fluorescence images were acquired from five randomly selected fields of view at ×100 magnification.

#### 3.4.5. Evaluation of Collagen and Elastin Synthesis—Col-F Assay

The effect of hydrogel extracts containing EvitC on collagen and elastin synthesis by hFB was evaluated after 24, 48 and 72 h of incubation. Following exposure, the culture medium was removed, and the cells were washed twice with PBS. Collagen and elastin were stained using the fluorescent probe Col-F (Biorbyt Ltd., Cambridge, UK) according to the manufacturer’s instructions. After incubation with the probe for 30 min at 37 °C, the cells were washed with PBS. Representative fluorescence images were acquired using an inverted fluorescence microscope (identified earlier). Fluorescence intensity was subsequently quantified using a Synergy HT microplate reader (identified earlier) at excitation and emission wavelengths of 490 and 520 nm, respectively. Results were expressed as a percentage of the untreated control. Measurements were performed in 6 technical replicates in three independent experiments (*n* = 3).

#### 3.4.6. Statistical Analysis

Quantitative biological data are presented as mean ± standard deviation (SD). For the XTT cytotoxicity assessment of free EvitC (Figure 7), cell viability was interpreted relative to the 70% threshold specified in ISO 10993-5. For the hydrogel extract XTT assay (Figure 9), the experiment was performed independently three times (*n* = 3), with six technical replicates per experimental condition. Technical replicates were averaged within each independent experiment before statistical analysis. At each incubation time (48 and 72 h), individual hydrogel extract groups were compared with the corresponding negative control (NC) using Dunnett’s multiple-comparison test. For the Col-F assay (Figure 11), the EvitC-containing hydrogel extract and the corresponding untreated control were compared using a paired two-tailed Student’s *t*-test. Statistical analyses were performed using OriginPro 2023 (OriginLab Corporation, Northampton, MA, USA). Differences were considered statistically significant at *p* < 0.05.

## 4. Conclusions

A radiation-crosslinked hydrogel based on poly(N-vinylpyrrolidone), poly(ethylene glycol) and agar was developed as a topical delivery platform for 3-O-ethyl-L-ascorbic acid (EvitC). Preliminary stability studies confirmed that EvitC exhibited markedly higher stability than native L-ascorbic acid under aqueous storage conditions, supporting its selection for hydrogel formulation.

The developed hydrogel exhibited favorable physicochemical properties, including high gel fraction, appropriate swelling behavior and satisfactory mechanical performance. Importantly, the radiation crosslinking process enabled simultaneous hydrogel formation and sterilization while preserving the biological activity of EvitC. A limitation of the proposed solution is the partial decomposition of the active component upon one-pot hydrogel synthesis, but to a significantly lesser extent than if irradiated in the solution. The hydrogel matrix provided additional protection of the active compound during radiation processing and subsequent storage, resulting in a stable product-like formulation with diffusion-driven release characteristics, which is distinct from the typical Fickan model due to the contribution of hydrogel swelling.

Biological evaluation demonstrated the cytocompatibility of both free 3-O-ethyl-L-ascorbic acid and EvitC-containing hydrogel extracts toward human dermal fibroblasts. Neither free EvitC nor the EvitC-containing hydrogel extracts induced cytotoxic effects. Prolonged exposure to the hydrogel extracts was associated with a moderate increase in extracellular matrix-associated fluorescence, although the contribution of the hydrogel matrix itself cannot be excluded.

The combination of radiation-induced hydrogel formation, preservation of EvitC stability after manufacturing, favorable physicochemical properties and cytocompatibility supports the potential of the developed material as a platform for the topical delivery of stabilized vitamin C derivatives. The proposed strategy may facilitate the development of sterile hydrogel-based formulations for skin care and post-procedure applications requiring the long-term stability of biologically active compounds.

Further research steps are required to fully evaluate the biological and translational potential of the developed platform. They should include evaluations of the skin penetration/permeation and in vivo performance of the developed hydrogel, as well as confirmation of the observed increase in ECM-associated Col-F fluorescence by specific molecular or biochemical analyses of extracellular matrix synthesis.

## Figures and Tables

**Table 1:** The effect of EvitC on the gel fraction (GF) and swelling degree at equilibrium (EDS) of PVP-based hydrogels produced by EB irradiation with 25 kGy dose (*n* = 3).

EvitC [%]	GF [%]	GF SD	EDS [%]	EDS SD
0	81.6	4.3	2860	430
0.05%	72.2	6.9	3170	420
0.10%	50.1	7.2	4040	620
0.25%	26.3	13.8	6700	1200
0.50%	0	-	0	-
* 0.50%	23.1	11.3	>10,000	>4000

* 10% PVP.

**Table 2:** The effect of EvitC on the tensile properties of PVP-based hydrogels produced by EB irradiation with 25 kGy dose (*n* = 3).

EvitC [%]	Elongation at Break [%]	SD	Max Strength [kPa]	SD	Elasticity Modulus [kPa]	SD
0	188.5	20.5	89.3	7.4	0.69	0.09
0.05%	255.2	43.6	64.9	10.4	0.64	0.13
0.10%	303.1	70.0	45.6	5.2	0.60	0.11
0.25%	nd **	-	nd	-	nd	-
* 0.50%	nd	-	nd	-	nd	-

* 10% PVP; ** nd—the material fragmented when handled, preventing reliable measurement.

**Table 3:** The data fit to the release models.

Model	R^2^	RMSE
Weibull, Q_∞_ = 50%	0.966	2.51
Weibull, Q_∞_ fitted	0.974	2.18
Korsmeyer–Peppas	0.732	5.47
Higuchi	0.830	5.57
Hixson–Crowell	0.729	7.03
Zero-order	0.648	8.01

**Table 4:** Change in essential parameters, characteristic for hydrogels with 0.1% EvitC after one-year storage (simulated) presented in absolute values and as a percentage with respect to initial values for hydrogels directly after synthesis, e.g., GF_12m_/GF_initial_ × 100%; EDS_12m_/EDS_initial_ × 100%. Thickness of the hydrogels for release experiments was ca. 4 mm. All data are the average of two independent measurements.

Parameter	Value After One Year of Aging	Percentage of the Initial Value [%]
GF	46.3 [%]	92.4
EDS	4300 [%]	106.4
Max elongation	279.5 [%]	92.2
Max strength	45.1 [kPa]	99.0
Elasticity modulus	57.9 [kPa]	96.5
EvitC release after 60 min	7.9 [% of total added]	112.9
EvitC release after 24 h	29.4% [% of total added]	130.7

## Data Availability

The data that support the findings of this study are available on request from the corresponding authors.

## Supplementary Materials

The following supporting information can be downloaded at: https://www.mdpi.com/article/10.3390/ijms27209028/s1. Refs [74,75,76,77,78,79,80,81,82,83] are cited in Supplementary Materials.
