# Supplementary material for: Delivery of 3-O-Ethyl-L-Ascorbic Acid from Radiation-Crosslinked Hydrogels to Promote Extracellular Matrix Production in Fibroblasts
Source: Int J Mol Sci. 2026 Oct 12;27(20):9028. doi: 10.3390/ijms27209028 (PMC13704985; doi:10.3390/ijms27209028)
Supplement: Supplementary file 1 [file ijms-27-09028-s001.zip › ijms-4464474-supplementary.pdf]

International Journal of
Molecular Sciences

MDPI

SUPPLEMENTARY MATERIALS

Delivery of 3-O-Ethyl-L-Ascorbic Acid from Radiation-Crosslinked Hydrogels to Promote Extracellular Matrix Production in Fibroblasts

Bozena Rokita [1,2,*], Agnieszka Adamus-Wlodarczyk [1,2], Alicja K. Olejnik [1,2], Renata Czechowska-Biskup [1,2], Beata Skibska [3], Slawomir Kadlubowski [1,2], Piotr Ulanski [1,2] and Radoslaw A. Wach [1,2,*]

[1] BioMatGel Sp. z o.o., Pienista 41F/11, 94-109 Lodz, Poland; slawomir.kadlubowski@p.lodz.pl (S.K.); piotr.ulanski@p.lodz.pl (P.U.)

[2] Institute of Applied Radiation Chemistry, Faculty of Chemistry, Lodz University of Technology, Wroblewskiego 15, 93-590 Lodz, Poland

[3] Department of Applied Pharmacy, Faculty of Pharmacy, Medical University of Lodz, Muszynskiego 1, 90-151 Lodz, Poland

* Correspondence: bozena.rokita@p.lodz.pl or bozena.rokita@biomatgel.pl (B.R.); radoslaw.wach@p.lodz.pl or radoslaw.wach@biomatgel.pl (R.A.W.)

S.1. Estimation of the upper limit of EvitC fraction degraded by radiation-induced reactions during hydrogel synthesis.

The most obvious effect of the irradiation process on EvitC is the partial degradation of the compound resulting from its reaction with •OH radicals. Fraction of EvitC degraded by •OH attack during the hydrogel synthesis has been estimated by stochastic kinetic simulations using Kinetiscope software, version 1.1.1328.x64 [SM1-SM3]. The rate constants of •OH reaction with EvitC, PVP, PEG and agar have been set as 1.1×10^{10}, 2×10^{8}, 1×10^{9} and 6×10^{8} M^{-1} s^{-1}, respectively. The value for EvitC is based on [48, 49]. For PVP and PEG the rate constant values stem from previously published results adjusted for molecular weight ([54] and [SM4], respectively), while the value for agar has been adopted from results reported for two other water-soluble polysaccharides, i.e., dextran [SM5] and chitosan [SM6] (Table S1). Simulation results indicate that approx. 11% of EvitC is expected to react with •OH during hydrogel synthesis in the studied system (Figure S1).

Table S1. Chemical reactions and their rate constants used to estimate the fraction of EvitC degraded via reaction with •OH radicals in the studied system during the radiation gel synthesis.

No.	Reaction	Rate constant	Reference
1.	H_2O => •OH	2.33×10^{-5} M s^{-1}	Based on the actual dose rate used in the experiments (83.3 Gy s^{-1}) and G(OH) = 2.8×10^{-7} mol J^{-1}.
2.	2 •OH => H_2O_2	5.5×10^{9} M^{-1} s^{-1}	[SM7]
3.	•OH + EvitC => •EvitC	1.1×10^{10} M^{-1} s^{-1}	[48, 49]

 https://doi.org/10.3390/ijms27209028

4.	•OH + PVP => •polymer	2×10^8 M^{-1} s^{-1}	[54]
5.	•OH + PEG => •polymer	1×10^9 M^{-1} s^{-1}	[SM4]
6.	•OH + agar => •polymer	6×10^8 M^{-1} s^{-1}	[SM5, SM6] – see text

Another possible pathway of partial destruction of EvitC during irradiation is the reaction of carbon-centered radicals located on PVP, PEG and agar molecules with EvitC. The most plausible mechanism would be an addition of such a radical to the C=C bond of the EvitC molecule. Such addition reactions are expected to have similar rate constants as propagation steps in free radical polymerization, being in the order of 10^3 – 10^4 M^{-1} s^{-1} [SM8, SM9]. While this is ca. 5 orders of magnitude lower than the expected rate constants of the competing polymer radical recombination reactions (10^8 – 10^9 M^{-1} s^{-1} [SM10]), attack of a carbon-centered radical on EvitC can still occur in our system with a considerable yield, since the polymer radical concentration during irradiation is expected to be much lower than the concentration of EvitC. An upper estimate of the EvitC decay yield via this mechanism has been made by kinetic simulations using Kinetiscope (Table S2), assuming the rate constant of a reaction between a polymer radical and EvitC of 1×10^4 M^{-1} s^{-1} as the upper limit, and recombination rate constant of polymer radicals of 1×10^8 M^{-1} s^{-1} as the lower limit. For completeness, mutual recombination of EvitC radicals as well as recombination of EvitC-derived radicals with polymer radicals have also been included at assumed values of 1×10^9 M^{-1} s^{-1} and 5×10^8 M^{-1} s^{-1}, respectively, but the two latter reactions have a negligible impact on EvitC decay. Simulation under such conditions indicates that up to 58 % of EvitC can be lost during irradiation, due to reactions with both •OH and carbon-centered polymer radicals (Figure S1).

Table A2. Chemical reactions and their rate constants used to estimate the fraction of EvitC degraded via reaction with •OH radicals and with polymer-based radicals in the studied system during the radiation gel synthesis.

No.	Reaction	Rate constant	Reference
1.	H_2O => •OH	2.33×10^{-5} M s^{-1}	Based on the actual dose rate used in the experiments (83.3 Gy s^{-1}) and G(OH) = 2.8×10^{-7} mol J^{-1}
2.	2 •OH => H_2O_2	5.5×10^9 M^{-1} s^{-1}	[SM7]
3.	•OH + EvitC => •EvitC	1.1×10^{10} M^{-1} s^{-1}	[48, 49]
4.	•OH + PVP => •polymer	2×10^8 M^{-1} s^{-1}	[54]
5.	•OH + PEG => •polymer	1×10^9 M^{-1} s^{-1}	[SM4]
6.	•OH + agar => •polymer	6×10^8 M^{-1} s^{-1}	[SM5, SM6] – see text
7.	•polymer + EvitC => adduct	1×10^4 M^{-1} s^{-1}	upper limit, based on [SM8, SM9] – see text

8.	2 •polymer => polymer product	1×10^{8} M^{-1} s^{-1}	lower limit, based on [SM10] – see text
9.	2 •EvitC => EvitC product	1×10^{9} M^{-1} s^{-1}	typical value
10.	•EvitC + •polymer => mixed product	5×10^{8} M^{-1} s^{-1}	typical value

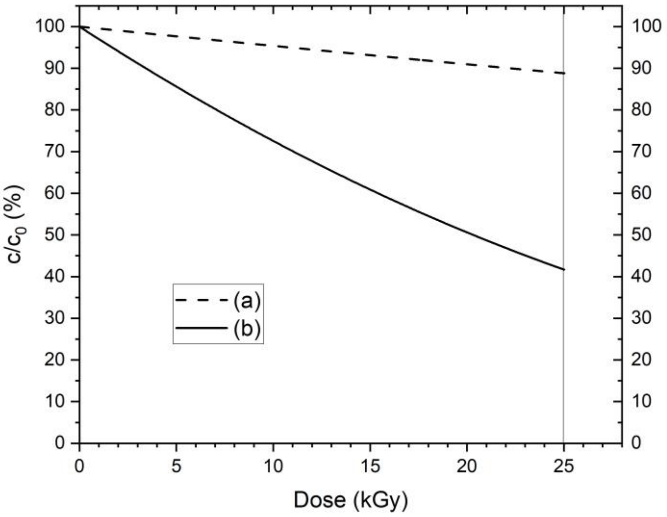

Figure S1. Simulated decay kinetics of EvitC in the irradiated system, assuming (a) degradation by •OH attack only and (b) degradation by reactions with both •OH radicals and radicals formed at PVP, PEG and agar. In case (b) reaction rate constants has been set to illustrate the "worst scenario", i.e., to show the upper estimate of the decay rate (see text). The total dose of 25 kGy (equivalent to the irradiation time of 300 s) corresponds to the experimental conditions of the radiation synthesis of hydrogels.

S.2. Sterility

The radiation method of sterilization is well established, and the radiation dose employed in the current synthesis approach was sufficient to sterilize the hydrogels. Sterility testing was conducted according to ISO 11737-2, and the results are presented in Figure S2. Hydrogel specimens were aseptically transferred into sterile bottles with universal growth medium and incubated in ca. 31°C up to 14 days. Results were negative, the medium remained transparent without signs of turbidity, i.e. no microorganism growth in medium means sterile samples.

https://doi.org/10.3390/ijms27209028

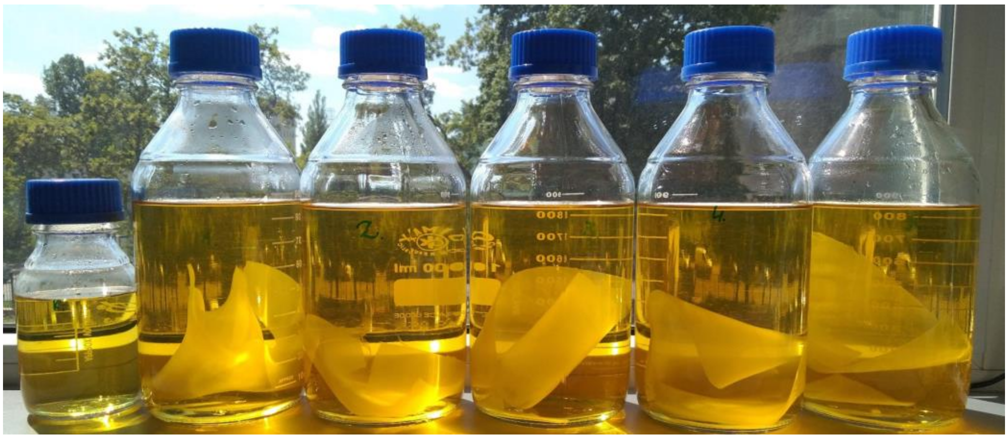

Figure S2. Hydrogels with EvitC after incubation for 14 days in growth medium; result: transparent; meaning: no microbial growth = sterile.

S.3. References

[SM1] https://www.hinsberg.net/kinetiscope/index.html (access date 29-09-2026)

[SM2] Gillespie, D.T. Stochastic simulation of chemical kinetics. Annual Review of Physical Chemistry 2007, 58, 35–55, doi: 10.1146/annurev.physchem.58.032806.104637

[SM3] Ulanski, P.; Bothe, E.; Hildenbrand, K.; von Sonntag, C. Free-radical-induced chain breakage and depolymerization of poly(methacrylic acid): equilibrium polymerization in aqueous solution at room temperature. Chemistry - A European Journal 2000, 6 (21), 3922–3934, doi: 10.1002/1521-3765(20001103)6:21<3922::AID-CHEM3922>3.0.CO;2-2

[48] Buettner, G.R.; Jurkiewicz, B.A. Catalytic metals, ascorbate and free radicals: Combinations to avoid. Radiation Research 1996, 145, 532, doi:10.2307/3579271

[49] Redpath, J.L.; Willson, R.L. Reducing compounds in radioprotection and radio-sensitization: Model experiments using ascorbic acid. International Journal of Radiation Biology and Related Studies in Physics, Chemistry and Medicine 1973, 23, 51–65, doi:10.1080/09553007314550051

[54] Bartoszek, N.; Ulański, P.; Rosiak, J.M. Reaction of a low-molecular-weight free radical with a flexible polymer chain: Kinetic studies on the OH + poly(N-vinylpyrrolidone) model. International Journal of Chemical Kinetics 2011, 43, 474–481, doi:10.1002/kin.20575

[SM44] Ulański, P.; Zainuddin; Rosiak, J.M. Pulse radiolysis of poly(ethylene oxide) in aqueous solution. I. Formation of macroradicals. Radiation Physics and Chemistry 1995, 46, 913–916, doi:10.1016/0969-806X(95)00291-5

[SM5] Szafulera, K.J.; Wach, R.A.; Ulański, P. Dextran methacrylate reactions with hydroxyl radicals and hydrated electrons in water: a kinetic study using pulse radiolysis. Molecules 2023, 28 (10), 4231, doi: 10.3390/molecules28104231

[SM6] Ulanski, P.; von Sonntag, C. OH-radical-induced chain scission of chitosan in the absence and presence of dioxygen. Journal of the Chemical Society. Perkin Transactions 2 2000, (10), 2022–2028, doi: 10.1039/b003952g

[SM7] Buxton, G.V.; Greenstock, C.L.; Helman, W.P.; Ross, A.B. Critical review of rate constants for reactions of hydrated electrons, hydrogen atoms and hydroxyl radicals ($^{\bullet}OH/O^{\bullet-}$) in aqueous solution. Journal of Physical and Chemical Reference Data 1988, 17 (2), 513–886, doi: 10.1063/1.555805

[SM8] Beuermann, S.; Buback, M. Rate coefficients of free-radical polymerization deduced from pulsed laser experiments. Progress in Polymer Science (Oxford) 2002, 27 (2), 191–254, doi: 10.1016/S0079-6700(01)00049-1

[SM9] Buback, M. Free-radical propagation rate coefficients. Canadian Journal of Chemical Engineering 2023, 101 (9), 5087–5098, DOI: 10.1002/cjce.24885

[SM10] Buback, M.; Russell, G.T. Detailed analysis of termination kinetics in radical polymerization. Polymer International 2023, 72 (10), 869–880, doi: 10.1002/pi.6501
